# Strategies for enhancing yield and quality of forage-grain dual-purpose ratoon rice: role of first-season density and nitrogen management

**DOI:** 10.3389/fpls.2025.1650539

**Published:** 2025-08-15

**Authors:** Yuyuan Ouyang, Guangyi Chen, Chuntao Yang, Conghua Zhu, Li Zhang, Wei Li, Hong Yang, Ziyu Li, Yao Zhang, Junqi Yu, Xi Luo, Xuyi Li, Tian Li

**Affiliations:** ^1^ College of Agronomy, Sichuan Agricultural University, Chengdu, China; ^2^ Crop Research Institute, Sichuan Academy of Agricultural Sciences, Chengdu, China; ^3^ Sichuan Pratacultural Technology Promotion Center, Chengdu, China; ^4^ Sichuan Provincial Key Laboratory of Green Germplasm Innovation and Genetic Improvement for Grain and Oil Crops, Chengdu, China

**Keywords:** silage rice, ratoon rice, planting density, nitrogen application rate, yield, quality

## Abstract

The “forage-grain dual-purpose” model helps ease land-use competition and supports high-yield, high-quality rice production. However, integrated strategies to simultaneously improve silage rice and ratoon rice yield and quality across both seasons require further systematic study. A two-year field study (2022–2023) was conducted using two widely cultivated indica hybrid rice cultivars, F You 498 (FY498) and Chuankangyou Simiao (CKYSM), in Southwest China. Treatments included three planting densities (D1: 16.7 × 10^4^; D2: 20.8 × 10^4^; D3: 27.8 × 10^4^ hills ha^−1^) and three nitrogen levels (N1: 150; N2: 225; N3: 300 kg ha^−1^) in the first season. Results showed that: Silage yield increased significantly with higher density and nitrogen input. FY498 reached the highest yield under D3N3 (48.46-57.60 t ha^−1^), while CKYSM performed best under D2N3 (45.39-50.93 t ha^−1^). Elevated density and nitrogen levels increased acid detergent fiber and neutral detergent fiber contents and reduced starch, indicating a decline in overall silage quality, though crude protein improved. Within the studied parameters, nitrogen application had a more pronounced influence on silage quality compared to planting density. Relative feed value was highest under D1N1 or D1N2, meeting national Grade II silage standards. In the ratoon season, increased density and nitrogen enhanced aboveground biomass, SPAD values, panicle number, and actual yield. However, higher density reduced leaf area index, and excess nitrogen decreased seed setting rate and 1000-grain weight. Maximum actual yields were observed under D2N3 or D3N2: FY498 (7.71-9.61 t ha^−1^) and CKYSM (6.49-9.00 t ha^−1^). Nitrogen application improved milling quality to some extent, while higher density negatively affected it. Both factors reduced appearance quality and RVA characteristics. Nutritional and safety quality varied significantly across treatments and cultivars. FY498 had high protein and low cadmium content under D1N3; CKYSM showed high starch and low cadmium under D2N3, indicating superior overall performance. In summary, D1N1 produced better silage and rice quality but lower yield. For higher overall productivity and safety, FY498 with D3N3 and CKYSM with D2N3 were optimal, despite moderate declines in quality traits. This new cultivation method may provide a beneficial option to balance silage rice and ratoon rice yield and quality.

## 1 Introduction

Enhancing rice production is critical for food security and can be achieved by expanding planting area, increasing yield per unit area, and improving the multiple cropping index. However, with accelerating urbanization and diminishing arable land, yield expansion through area increase has become unsustainable (Liu and Sun, 2008). Ratoon rice, which regrows from dormant buds on rice stubble after the first crop harvest, enables “one planting, two harvests” and offers an efficient alternative (Zhang et al., 2020; Yang et al., 2022). Compared with single-season rice, ratoon rice increases production costs by 35-48% but yields 72-129% higher net profit. Compared with double-season rice, it reduces costs by 32-42% and nearly doubles profitability (Yuan et al., 2019; Wu et al., 2019). Sichuan Province, the birthplace of ratoon rice in China, leads the country in planting area and yield. The “mid-season rice + ratoon rice” system has evolved into a highly efficient model that combines dual harvests, labor-saving practices, and high grain quality. It improves resource use efficiency, enhances the multiple cropping index, and serves as a key approach to stabilizing grain production and improving rice quality in the region (Xiong et al., 2000; Zhang et al., 2012).

Meanwhile, forage supply is a bottleneck in livestock development. Sichuan requires approximately 39.7 million tons of silage annually, but local production accounts for less than 2%, leaving a massive 98% supply gap (Zhang et al., 2008a; Zhang et al., 2023a). In this context, integrating forage production into existing cropping systems is crucial. The “forage-grain dual-purpose” model-where green biomass is harvested during early growth and grain is collected later-offers a promising strategy to enhance land-use efficiency (Zhang et al., 2023b). Our previous research showed that under a “first-season forage + ratoon rice” model, forage dry matter crude protein content reached 9.08-13.10%, meeting first-grade silage standards. The ratoon rice also exhibited superior eating quality compared to conventional single-season rice. Moreover, this system improved net income by over 7500 CNY per hectare, demonstrating both economic and ecological benefits (Chen et al., 2022; Wu et al., 2024). It can reduce reliance on imported feed like soybean meal, corn, and alfalfa, thus supporting feed security and sustainable livestock development (Fu, 2008; Zhang et al., 2008b).

Globally, Japan has pioneered silage rice breeding since 1986 and developed multiple specialized cultivars (Sakai et al., 2003). Research there has systematically explored how cutting time, stubble height, planting density, and nitrogen application rate affect forage yield and regrowth (Nakano and Morita, 2008; Nakano et al., 2009, 2019). Among these factors, planting density and nitrogen input are key agronomic practices influencing both yield and quality. Optimal density improves canopy structure and light use efficiency. Within the planting density range of 27.0 to 39.0 × 10^4^ hills ha^−1^ for ratoon rice, both first-season and ratoon-season yields initially increased and then decreased with increasing density. There were no significant differences in first-season yield among the different density treatments, while significant differences were observed in ratoon-season yield (Wu and Wu, 2013). Excessively low or high densities reduce tillering, hinder ratoon bud sprouting, or increase disease risk (Lv et al., 2010; Tang et al., 2014). Nitrogen application is equally critical, directly affecting vegetative growth, photosynthesis, and dry matter accumulation (Wang et al., 2021). Moderate nitrogen input in the mid-to-late main crop stage enhances leaf enzyme activity, chlorophyll content, and net photosynthetic rate, increasing first-season yield with limited impact on the ratoon crop (Yang et al., 2009). In the first season, under the same total fertilizer application rate, increasing the proportion of panicle fertilizer improved dry matter and nitrogen accumulation, thereby enhancing first-season yield. The highest yield was achieved when the ratio of basal and tiller fertilizer to panicle fertilizer was 6:4 (Chen et al., 2010). Although the nitrogen management in the first season had no significant effect on ratoon-season yield, some studies have indicated that higher panicle fertilizer input also improves source-sink relationships, benefiting both seasons’ yields (Huang et al., 2022).

Although previous studies have addressed the effects of planting density and nitrogen rates on rice performance, few have systematically examined their interactions in the dual-purpose system of “silage rice + ratoon rice.” Besides, crop management practices, such as irrigation, weed and pest control still need additional in-depth and systematic research. How to ensure high yield and quality of silage rice in the first season while maintaining the regenerative capacity and yield formation of ratoon rice in the second season is a core scientific question that urgently needs to be addressed for the coordinated development of food and forage production. Therefore, this study used dual-purpose rice cultivars suitable for the southern double-cropping region to explore the effects of three planting densities and three nitrogen levels under the innovative “forage-grain dual-purpose” cultivation model. We aimed to evaluate their impacts on yield and quality of silage rice and ratoon rice, and to provide theoretical and technical support for developing a high-efficiency, stable forage-grain integrated cultivation system.

## 2 Materials and methods

### 2.1 Experimental site and materials

This experiment was conducted in 2022–2023 at the experimental base of the Grain-Economic Complex Expert Compound in Mianzhu, Sichuan Province (N 31°15′, E 104°13′). The experiment site comprised loam soil, and the previous crop was wheat. Prior to the establishment of the field experiment, soil samples from the topsoil layer (0-0.20 m) were analyzed. The national meteorological monitoring station (CAWS600, China Huayun Meteorological Technology Group Co., Ltd., Beijing, China) was used to record the average daily temperature, maximum temperature and rainfall in the rice growing season. The complete data of top soil layer and climate data is available in **Supplementary Table S1** and **Supplementary Figure S1**, respectively.

The experiment utilized two indica three-line hybrid rice cultivars-F You 498 (Rice Research Institute of Sichuan Agricultural University) and Chuankangyou Simiao (Crop Research Institute of the Sichuan Academy of Agricultural Sciences)-both of which were identified in previous studies as having strong regeneration capacity and are widely cultivated in the southwest regions. The average growth periods of FY498 and CKYSM were 155.2 and 149.4 days, respectively.

### 2.2 Experimental design

An orthogonal experimental design was adopted with two factors in two years, using a randomized complete block design with three replicates. Nine treatments were established by the complete combination of three planting densities (16.7 × 10^4^, 20.8 × 10^4^ and 27.8 × 10^4^ hills ha^-1^, denoted as D1, D2 and D3, respectively) and three N application rates (150, 225 and 300 kg ha^-1^, denoted as N1, N2 and N3, respectively).

Seeds were sown on 15 March, and the seedlings were transplanted on 15 April, and silage rice was harvested 15 days after full heading, and ratoon rice was harvested on 8 Oct 2022 and 11 Oct 2023, respectively. The area of each test plot was 30.0 m^2^, and the planting density was at the treatment densities specified above with four seedlings per hill. Urea (N, 46.4%) was used as the N source, superphosphate (P_2_O_5_, 12.0%) was used as the phosphorus (P) source, and potassium chloride (K_2_O, 60.0%) was used as the K source.

The total nitrogen fertilizer was allocated between the silage and ratoon rice seasons at a ratio of 6:4. In the silage season, N fertilizer was applied at a 5:3:2 ratio of basal fertilizer/tillering fertilizer/panicle fertilizer. Among them, the basal fertilizer was applied one day before transplanting and sowing, the tillering fertilizer was applied 10 days after transplanting (at the fifth leaf stage for direct seeding), and the panicle fertilizer was applied at the third stage of young panicle differentiation. P (75 kg ha^-1^) and K (150 kg ha^-1^) fertilizers were applied one time as basal fertilizers. In the ratoon season, N fertilizer (promoting tiller growth fertilizer) was applied during rewatering following forage harvest. The aboveground portion of the silage-season rice was harvested as forage 15 days after full heading, leaving a stubble height of 15 cm. Immediately after removing the straw, the field was rewatered to prevent stubble desiccation due to high temperatures and to suppress excessive weed germination caused by shallow water levels. For the fertilizer treatments, ridges with plastic film were used for separation, and protection lines were established between the treatment blocks to ensure the isolation of the experimental plots. Field management, including the prevention and control of pests and weeds, was conducted according to the local cultural practices.

### 2.3 Measurements and methods

#### 2.3.1 Dry matter accumulation

At heading stage and maturity stage, 6 representative plants were selected according to the average tillering number, and the plants were cleaned and separated into 3 parts: stem-sheath, leaf, and panicle. All the samples were blanched at 110°C for 30 min, transferred to 80°C for drying to a constant weight, and weighed dry matter (DM) accumulation.

#### 2.3.2 Relative chlorophyll content and leaf area index

10 flag leaves with the same growth were selected 15 days after full heading. The relative chlorophyll content was measured with a portable chlorophyll meter (SPAD-502, Konica Minolta Holdings Inc., Tokyo, Japan). Six points on each leaf were chosen from measurement with SPAD. These comprised three pairs of points on both sides of the midrib were sampled near the leaf tip, in the middle of the leaf, and near the leaf bottom. The mean of these six SPAD values was recorded. The leaf area index (LAI) was measured with a portable leaf area meter (LI-3100, LI-COR Inc., NE, USA).

#### 2.3.3 Ratoon rice yield and yield components

At maturity stage, 60 holes were selected from each plot to investigate the average tillering number and then 6 representative plants were selected, and the panicle number (PN), spikelet number per panicle (SP), seed setting rate (SR), and 1000-grain weight (GW) were investigated. Finally, the actual yield (AY) was calculated and adjusted to a moisture content of 13.5%.

#### 2.3.4 Ratoon rice quality

At harvest, 10 holes of plants from each plot were sampled randomly and allowed to dry naturally in the sun to assess rice milling quality and appearance quality after the material was stored at room temperature for 3 months. The milled rice was crushed and sieved through a 100-mesh screen for the measurements of rapid visco-analyzer (RVA). Another 10 holes of plants from each plot were sampled randomly and blanched at 110°C for 30 min, transferred to 80°C for drying to a constant weight, and weighed, after which the brown rice was crushed and sieved through a 100-mesh screen for the measurements of protein content, total starch content (TS), amylose content (AC) and cadmium content (Cd).

About 130.0 g rice grains were processed by using a rice huller (JLG-2118, Taizhou Food Instrument Co., Ltd., Zhejiang, China) to obtain brown rice (BR). The brown rice was polished by using a rice milling machine (JNMJ-3, Taizhou Food Instrument Co., Ltd., Zhejiang, China) to obtain milled rice (MR). In order to obtain head-milled rice (HR), grain with a length longer than 3/4 of its total length was separated from the milled rice by using a broken rice separator (FQS-13X20, Taizhou Food Instrument Co., Ltd., Zhejiang, China). The brown rice, milled rice, and head-milled rice are expressed as percentages of the total grain weight. The chalkiness rate (CR) and chalkiness degree (CD) were determined using a grain appearance analyzer (JMWT12, Dongfu Jiuheng Instrument Technology Co., Ltd., Beijing, China).

A 3.00 g sample and 25.0 mL of distilled water were added to a test tube. Pasting properties were measured by using a rapid visco-analyzer device (3-D, Newport Scientific, Sydney, Australia) and analyzed with Thermal Cycle for Windows software. Viscosity values were measured in centipoise (RVU).

The protein content was measured based on the total N content of milled rice with a conversion index of 5.95 via the Kjeldahl method.

The total starch content was measured by the anthrone colorimetric method (Gao, 2006). 0.1 g rice flour sample was extracted by 5.0 mL 80% ethanol at 80°C for 30 min. After repeated extraction and centrifugation (6000 r min^-1^ for 5 min) for three times, the supernatant (testing solution) was combined and the volume was adjusted to 100 mL. Aliquots (2 mL) of the extract were analyzed for sucrose and soluble sugar content. The remaining precipitate was used for the determination of total starch content.

The amylose content was measured by the iodine reagent method (Chen et al., 2020). 10 mL 0.5 mol L^-1^ KOH was added to 1.0 g rice flour sample, followed by the addition of 5.0 mL 1.0 mol L^-1^ HCl and 0.5 mL iodine reagent. After adjustment to 100 mL with distilled water, the absorbance was measured at 620 nm after 20 min by scanning the iodine absorption spectrum from 400 to 900 nm with a spectrophotometer (Ultrospec 6300 pro, Amershan Biosciences, Cambridge, Sweden). The values were converted to amylose content by reference to a standard curve prepared from rice.

A 0.200 g sample and 10.0 mL of mixed acid solution (V_HNO3_/V_HClO4_ = 4:1) were added to a 50 mL conical flask. Allow the mixture to stand overnight. Subsequently, digest the sample at 220°C until the solution becomes clear. When the solution is nearly evaporated to dryness, terminate the digestion and allow it to cool naturally. Rinse the residue with ultrapure water and dilute to a final volume of 50 mL in a volumetric flask. Filter the solution through a 0.45 μm membrane into a 10 mL centrifuge tube. The cadmium content in the resulting solution is then determined using inductively coupled plasma mass spectrometry (Agilent7700x, Agilent Technologies Co., Ltd., Santa Clara, CA, USA).

#### 2.3.5 Silage rice yield and quality

15 d after full heading, the border rows of each plot were removed, and all aboveground biomass within the plots was harvested to determine fresh forage yield. The stubble height of 15 cm was maintained.

At the time of forage harvest, 10 representative plants were selected from each plot. The plant samples were cut into segments approximately 2 cm in length. Stems, leaves, and panicles were thoroughly mixed and packed into silage bags, vacuum-sealed, and allowed to ferment naturally at room temperature for 60 d. After fermentation, quality parameters of the silage were analyzed. Silage rice quality assessment was conducted by Hangzhou Aiko Testing Technology Co., Ltd. Evaluation of first-season silage rice quality followed the national standard GB/T 25882–2010 of the People’s Republic of China, Classification of Silage Corn Quality. Relative feed value (RFV) was calculated using the following equations:


Dry matter intake (%)=120/Neutral detergent fiber (NDF) content

Digestible dry matter (%)=88.9-0.779×Acid detergent fiber (ADF) content

Relative feed value=(Dry matter intake×Digestible dry matter)/1.29

### 2.4 Statistical analysis

Analysis of variance (ANOVA) was used to analyze the data, and means were compared based on the least significant difference (LSD) test at a 0.05 probability level using SPSS 21.0 (Statistical Product and Service Solutions Inc., Chicago, IL, USA). Figures were plotted using Origin Pro 2022 (OriginLab, Northampton, MA, USA). The differences of silage rice yield, LAI, SPAD value and dry matter accumulation are available in **Supplementary Tables S2**, **S3**, respectively.

## 3 Results

### 3.1 Silage rice yield

**Figure 1:**
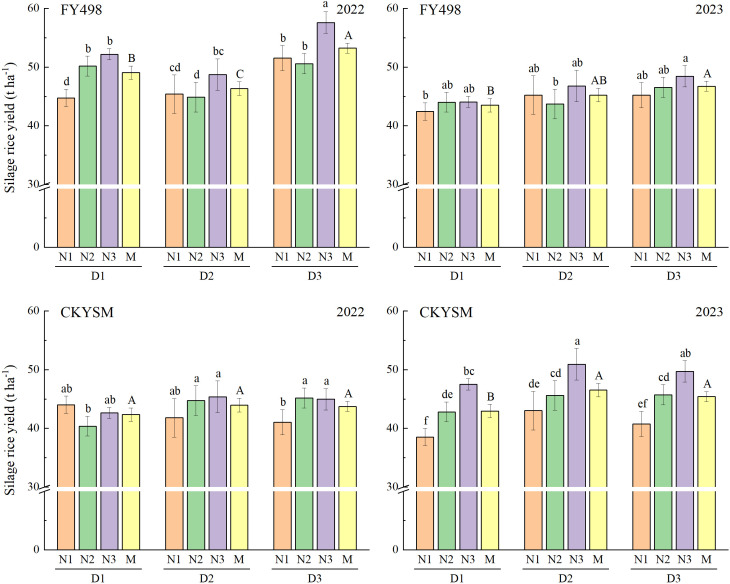
Effects of planting density and nitrogen application rate combinations on silage rice yield. D1, D2 and D3 refer to the different planting density treatments (16.7 × 10^4^, 20.8 × 10^4^ and 27.8 × 10^4^ hills ha^-1^, respectively). N1, N2 and N3 refer to the different nitrogen fertilizer treatments (150, 225 and 300 kg ha^-1^, respectively). M represents the average of different nitrogen fertilizer levels under the same planting density. Different lowercase letters mean the significant difference of the different combined application of D and N levels at *p*< 0.05. Different uppercase letters mean the significant difference of different average D levels at *p*< 0.05. The data presented are the mean ± standard deviation, *n* = 3.

Silage rice yield of both FY498 and CKYSM exhibited an overall increasing trend with higher planting density and nitrogen application (**Figure 1**). An exception was observed for CKYSM in 2022, where silage rice yield slightly decreased under low planting density and low nitrogen input. Yield improvement in FY498 was more pronounced at high planting density, whereas CKYSM showed the greatest response under medium planting density. ANOVA results revealed that both planting density and nitrogen application had highly significant effects on silage rice yield in both cultivars (**Supplementary Table S2**).

Across the two growing seasons, the highest silage rice yield for FY498 was obtained under the D3N3 treatment, reaching 57.60 t ha^-1^ in 2022 and 48.46 t ha^-1^ in 2023. For CKYSM, the D2N3 treatment produced the highest yields-45.39 t ha^-1^ in 2022 and 50.93 t ha^-1^ in 2023. Compared to the lowest treatment, FY498 showed yield increases of 28.69% in 2022 and 14.73% in 2023, while CKYSM yields increased by 12.41% and 32.22%, respectively.

Overall, increasing planting density and nitrogen input in the first-season crop effectively enhanced silage rice yield. Particularly, treatments with medium to high planting density combined with high nitrogen application (D2N3 or D3N3) consistently achieved significant yield gains.

### 3.2 Silage rice quality

Silage rice quality is commonly assessed based on the contents of CP, ADF, NDF, and starch. Higher CP and starch levels, alongside lower ADF and NDF levels, are indicative of superior quality.

With increasing planting density and nitrogen application, ADF and NDF contents in both FY498 and CKYSM generally increased, whereas starch content decreased. CP content exhibited a clear increasing trend with nitrogen input but showed no consistent response to changes in planting density (**Table 1**). ANOVA results revealed that planting density had a highly significant effect on ADF content, while nitrogen application had significant or highly significant effects on all four quality parameters across both cultivars.

**Table 1:** Effects of planting density and nitrogen application rate combinations on silage rice quality.

Treatment		CP (%)		ADF (%)		NDF (%)		Starch (%)		RFV	
		FY498	CKYSM	FY498	CKYSM	FY498	CKYSM	FY498	CKYSM	FY498	CKYSM
D1	N1	10.30 ± 0.29c	11.10 ± 0.68cd	23.19 ± 1.33c	25.22 ± 1.49cd	49.32 ± 1.06c	52.07 ± 2.52cd	28.90 ± 1.31a	26.10 ± 1.07a	133.65 ± 3.57a	123.91 ± 5.84b
	N2	11.60 ± 0.27ab	12.83 ± 0.84ab	23.75 ± 0.76c	24.10 ± 1.36d	51.41 ± 0.93abc	48.83 ± 1.46d	25.98 ± 0.45bc	23.37 ± 1.01bc	127.41 ± 2.73abc	133.63 ± 2.85a
	N3	11.98 ± 0.65ab	13.82 ± 0.49a	24.46 ± 1.55bc	26.19 ± 0.89bcd	52.53 ± 2.26abc	55.13 ± 2.93abc	24.79 ± 0.49c	22.10 ± 1.17c	123.87 ± 6.65bcd	115.75 ± 4.94bcd
	Mean	11.29 ± 0.23A	12.58 ± 0.42A	23.80 ± 0.31B	25.17 ± 0.98B	51.09 ± 0.77B	52.01 ± 1.68C	26.56 ± 0.4A	23.86 ± 0.63A	128.31 ± 1.79A	124.43 ± 2.86A
D2	N1	10.41 ± 0.53c	12.23 ± 0.98bcd	23.43 ± 1.14c	26.77 ± 1.52bc	50.12 ± 2.34bc	54.08 ± 2.49bc	27.48 ± 1.85ab	24.68 ± 0.91ab	131.27 ± 4.61ab	117.25 ± 6.81bcd
	N2	11.20 ± 0.71bc	12.50 ± 0.60ab	24.02 ± 1.58bc	21.73 ± 1.00e	52.20 ± 3.21abc	54.70 ± 3.76abc	26.69 ± 1.16bc	23.86 ± 0.73bc	125.37 ± 7.58abc	122.74 ± 7.43bc
	N3	11.92 ± 0.68ab	12.41 ± 0.38abc	25.41 ± 1.75abc	28.47 ± 1.34ab	53.02 ± 1.70ab	56.23 ± 1.11abc	25.00 ± 1.11c	21.93 ± 1.16c	121.30 ± 3.47cd	110.41 ± 3.17de
	Mean	11.18 ± 0.36A	12.38 ± 0.10A	24.28 ± 0.64B	25.65 ± 1.09B	51.78 ± 1.25AB	55.01 ± 1.50B	26.39 ± 0.32A	23.49 ± 0.50A	125.98 ± 3.83A	116.80 ± 2.33B
D3	N1	11.05 ± 0.73bc	11.03 ± 0.47d	25.70 ± 1.38abc	30.10 ± 2.08a	51.95 ± 1.51abc	58.05 ± 1.36ab	26.46 ± 1.20bc	22.63 ± 1.58bc	123.38 ± 1.67bcd	104.91 ± 2.39e
	N2	11.69 ± 0.57ab	13.49 ± 1.01ab	26.65 ± 1.51ab	26.62 ± 1.27bc	53.35 ± 2.05ab	56.14 ± 3.72abc	25.21 ± 1.45c	22.30 ± 1.14c	118.94 ± 5.29cd	113.29 ± 8.04cde
	N3	12.52 ± 0.62a	13.30 ± 1.08ab	27.95 ± 0.67a	29.27 ± 1.01a	53.90 ± 1.07a	59.16 ± 2.47a	24.76 ± 0.47c	22.05 ± 1.01c	115.89 ± 2.76d	104.03 ± 3.67e
	Mean	11.68 ± 0.62A	12.60 ± 0.75A	26.76 ± 0.82A	28.66 ± 1.01A	53.06 ± 0.84A	57.78 ± 0.88A	25.48 ± 0.78B	22.33 ± 0.50B	119.41 ± 1.13B	107.41 ± 0.82C
F-value	D	2.640ns	0.267ns	11.225**	19.619**	2.626ns	12.983**	2.408ns	4.969*	9.647**	23.156**
	N	17.208**	15.477**	3.809*	23.106**	4.959*	5.129*	13.768**	11.666**	9.480**	13.955**
	D × N	0.293ns	3.477*	0.111ns	3.649*	0.097ns	0.927ns	1.256ns	2.291ns	0.078ns	0.687ns

D1, D2 and D3 refer to the different planting density treatments (16.7 × 10^4^, 20.8 × 10^4^ and 27.8 × 10^4^ hills ha^-1^, respectively). N1, N2 and N3 refer to the different nitrogen fertilizer treatments (150, 225 and 300 kg ha^-1^, respectively). CP, ADF, NDF and RFV represent crude protein, acid detergent fiber, neutral detergent fiber and relative feed value, respectively. Different lowercase letters followed the values in the same column mean the significant difference of the different combined application of D and N levels at *p*< 0.05. Different uppercase letters mean the significant difference of different average D levels at *p*< 0.05. ANOVA *p* values and symbols were defined as: * *p*< 0.05; ** *p*< 0.01; ns: *p* > 0.05, ns means non-significant. The data presented are the mean ± standard deviation, *n* = 3.

According to the national standard for silage maize quality classification (GB/T 25882-2010), CP contents under all treatments for both cultivars exceeded the Grade I threshold (≥7%), while starch content met the Grade II standard (≥20%). For FY498, both ADF and NDF levels consistently met the Grade III standard (ADF ≤ 23%, NDF ≤ 55%) under all treatments. For CKYSM, these thresholds were only met under medium or lower levels of planting density and nitrogen application.

Overall, increased planting density and nitrogen input tended to reduce silage rice quality, primarily due to elevated ADF and NDF contents and decreased starch content. The highest RFV for FY498 and CKYSM was observed under D1N1 and D1N2, respectively. Under these conditions, FY498 showed a CP content of 10.30%, ADF of 23.19%, NDF of 49.32%, and starch of 28.90%, while CKYSM had a CP content of 12.83%, ADF of 24.10%, NDF of 48.83%, and starch of 23.37%. These values indicate that both cultivars achieved the Grade II standard for high-quality forage (CP ≥ 7%, ADF ≤ 26%, NDF ≤ 50%, starch ≥ 20%).

### 3.3 Dry matter accumulation of ratoon rice

**Figure 2:**
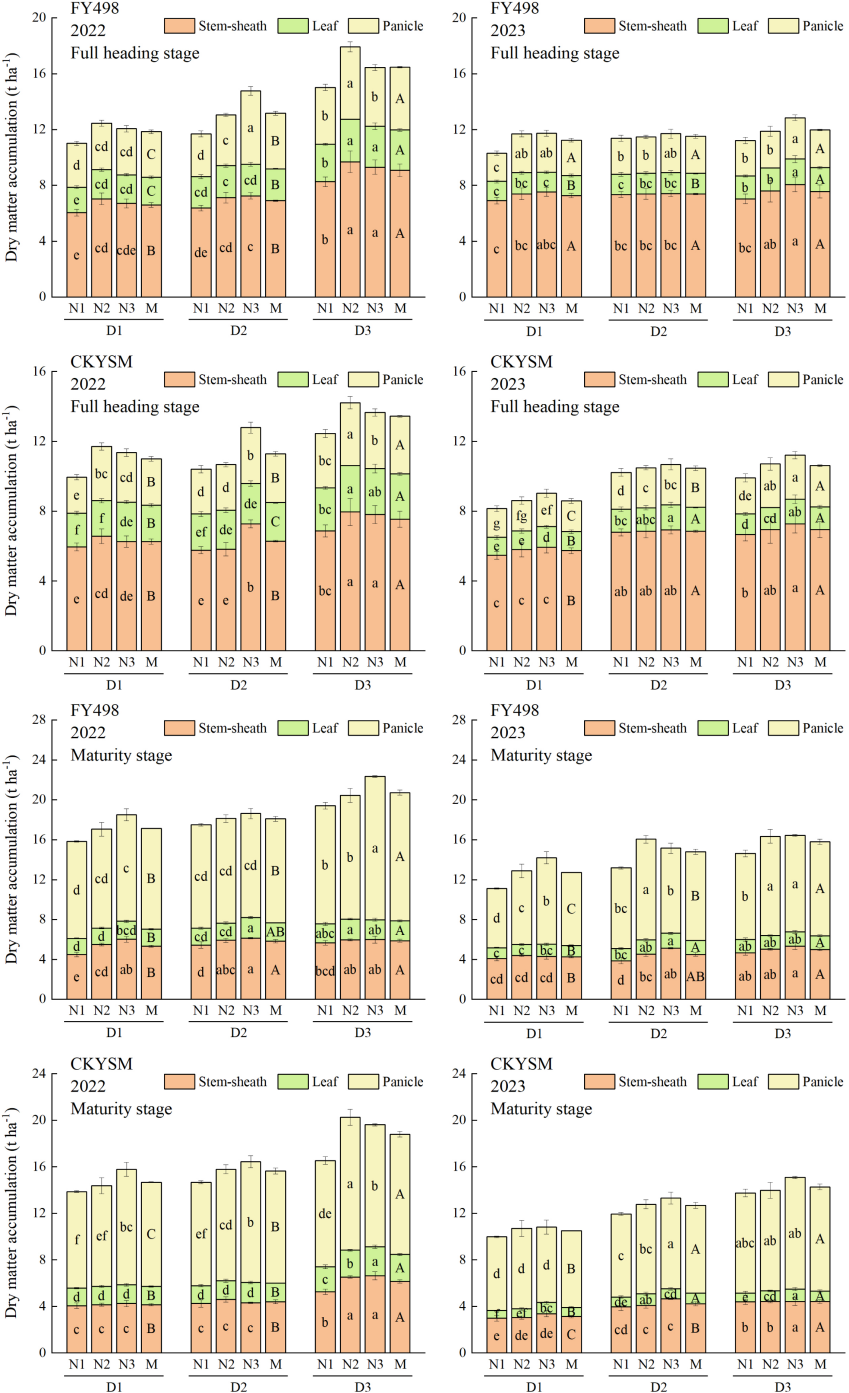
Effects of planting density and nitrogen application rate combinations on silage rice dry matter accumulation at full heading stage (above) and maturity stage (below). D1, D2 and D3 refer to the different planting density treatments (16.7 × 10^4^, 20.8 × 10^4^ and 27.8 × 10^4^ hills ha^-1^, respectively). N1, N2 and N3 refer to the different nitrogen fertilizer treatments (150, 225 and 300 kg ha^-1^, respectively). M represents the average of different nitrogen fertilizer levels under the same planting density. Different lowercase letters mean the significant difference of the different combined application of D and N levels at *p*< 0.05. Different uppercase letters mean the significant difference of different average D levels at *p*< 0.05. The data presented are the mean ± standard deviation, *n* = 3.

Increasing planting density in the first season significantly enhanced the accumulation of aboveground DM in both rice cultivars, including stem-sheath, leaf, and panicle. Under medium to high planting densities, additional nitrogen input (D3N2 or D3N3) further promoted DM accumulation (**Figure 2**).

As planting density increased, the DM accumulation of stem-sheath, leaf, panicle, and total aboveground biomass at both heading and maturity stages consistently increased in both FY498 and CKYSM. In contrast, DM accumulation showed a variable response to nitrogen input, with most treatments following an increasing or first increasing and then decreasing trend (N2 > N3 > N1). ANOVA results revealed that both planting density and nitrogen application had highly significant effects on DM accumulation in the aboveground organs. The interaction between planting density and nitrogen application also had significant or highly significant effects on total DM accumulation (**Supplementary Table S3**).

The highest total DM accumulation at both heading and maturity stages was observed under D3N2 or D3N3. Compared with the lowest treatment, FY498 showed increases in total DM accumulation at heading by 62.70% in 2022 and 24.64% in 2023, while CKYSM showed increases of 43.06% and 37.71%, respectively. At maturity stage, FY498 exhibited increases of 40.95% in 2022 and 47.88% in 2023, whereas CKYSM reached increases of 46.18% and 51.10%, respectively.

### 3.4 Physiological characteristics of ratoon rice

**Figure 3:**
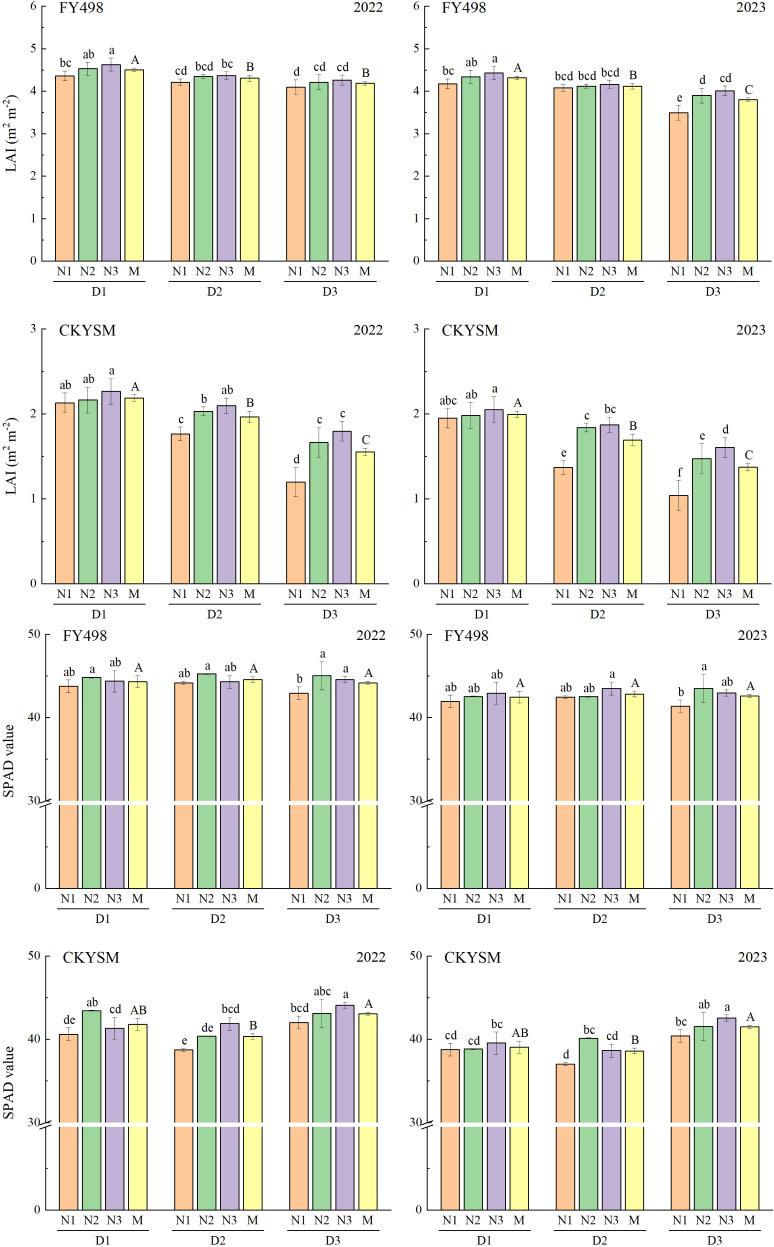
Effects of planting density and nitrogen application rate combinations on silage rice flag leaf LAI and SPAD value 15d after full heading. D1, D2 and D3 refer to the different planting density treatments (16.7 × 10^4^, 20.8 × 10^4^ and 27.8 × 10^4^ hills ha^-1^, respectively). N1, N2 and N3 refer to the different nitrogen fertilizer treatments (150, 225 and 300 kg ha^-1^, respectively). M represents the average of different nitrogen fertilizer levels under the same planting density. Different lowercase letters mean the significant difference of the different combined application of D and N levels at *p*< 0.05. Different uppercase letters mean the significant difference of different average D levels at *p*< 0.05. The data presented are the mean ± standard deviation, *n* = 3.

As planting density increased, LAI of both FY498 and CKYSM exhibited a decreasing trend, whereas changes in SPAD values were inconsistent (**Figure 3**). In contrast, increasing nitrogen application resulted in a general increase in LAI for both cultivars, while SPAD values showed either a continuous increase or first increase and then decrease trend, typically following the trend of N1< N3< N2. ANOVA results revealed that planting density had a highly significant effect on LAI, while nitrogen application had highly significant effects on both LAI and SPAD value across the two cultivars (**Supplementary Table S2**).

For FY498, the highest LAI was observed under D1N3, and the highest SPAD values were recorded under D2N2 or D2N3. For CKYSM, the maximum LAI and SPAD values were obtained under D1N3 and D3N3, respectively. Compared to other treatments, the maximum LAI of FY498 increased by 12.93% in 2022 and 13.59% in 2023, while the maximum SPAD values increased by 5.40% and 5.20%, respectively. For CKYSM, the maximum LAI increased by 88.33% in 2022 and 97.12% in 2023, and SPAD values increased by 13.84% and 14.99%, respectively.

In summary, increasing planting density in the first season tends to reduce LAI, whereas increasing nitrogen input can enhance both LAI and SPAD value within a certain range. Specifically, low planting density combined with high nitrogen input (D1N3) was effective in improving LAI, while medium to high planting densities coupled with moderate to high nitrogen input (D2N2 or D2N3) contributed to higher SPAD values.

### 3.5 Ratoon rice yield and yield components

Increasing planting density and nitrogen application in the first season significantly improved AY and PN in ratoon rice (**Tables 2**, **3**). High yields were achieved either under moderate planting density combined with high nitrogen input (D2N3) or under high planting density with moderately increased nitrogen input (D3N2).

**Table 2:** Effects of planting density and nitrogen application rate combinations on ratoon rice yield of FY498.

Treatment		PN (×10^4^ ha^-1^)		SP		SR (%)		GW (g)		AY (t ha^-1^)	
		2022	2023	2022	2023	2022	2023	2022	2023	2022	2023
D1	N1	506.67 ± 11.55d	260.00 ± 10.00e	71.78 ± 2.64e	56.51 ± 1.02d	64.79 ± 1.60a	72.08 ± 0.63ab	30.25 ± 0.51abc	31.30 ± 0.39a	8.65 ± 0.38e	5.92 ± 0.34d
	N2	516.67 ± 5.77cd	273.33 ± 5.77de	86.49 ± 2.68cd	62.54 ± 1.90bc	61.39 ± 1.38b	66.23 ± 1.11d	30.10 ± 0.16abc	31.28 ± 0.48a	8.80 ± 0.57de	6.27 ± 0.07cd
	N3	523.34 ± 11.55cd	310.00 ± 17.32b	87.00 ± 3.49cd	73.56 ± 2.45a	57.42 ± 1.53c	65.05 ± 3.20d	30.04 ± 0.13bc	30.72 ± 0.17a	9.11 ± 0.17bcde	6.44 ± 0.58cd
	Mean	515.56 ± 1.92C	281.11 ± 5.09C	81.76 ± 1.61C	64.20 ± 0.52B	61.20 ± 1.10A	67.79 ± 0.88B	30.13 ± 0.16A	31.10 ± 0.12A	8.85 ± 0.29B	6.21 ± 0.26B
D2	N1	510.00 ± 10.00d	286.67 ± 5.77cd	85.41 ± 4.57cd	62.03 ± 3.49bc	59.18 ± 1.09bc	72.03 ± 0.92ab	30.74 ± 0.36ab	31.62 ± 0.59a	8.91 ± 0.25cde	6.75 ± 0.57bc
	N2	540.00 ± 17.32c	306.67 ± 11.55bc	90.32 ± 5.86bc	66.75 ± 2.76b	57.82 ± 1.10c	69.70 ± 1.40bc	30.34 ± 1.08abc	31.25 ± 0.74a	9.15 ± 0.08abcd	7.13 ± 0.28ab
	N3	536.67 ± 11.55c	323.33 ± 23.09ab	100.58 ± 5.52a	75.14 ± 1.45a	57.16 ± 0.44c	67.20 ± 2.68cd	29.63 ± 0.70c	31.21 ± 0.79a	9.24 ± 0.3abcd	7.71 ± 0.36a
	Mean	528.89 ± 6.94B	305.56 ± 6.94B	92.10 ± 0.25A	67.97 ± 2.38A	58.05 ± 0.6B	69.65 ± 0.88AB	30.24 ± 0.39A	31.36 ± 0.28A	9.10 ± 0.18B	7.20 ± 0.31A
D3	N1	650.00 ± 10.00b	286.67 ± 11.55cd	82.07 ± 1.05d	61.46 ± 3.85c	64.39 ± 1.76a	74.26 ± 1.80a	31.11 ± 0.19a	31.63 ± 0.74a	9.38 ± 0.16abc	7.38 ± 0.21ab
	N2	660.00 ± 10.00b	333.33 ± 15.28a	87.40 ± 3.35cd	66.32 ± 3.32bc	61.60 ± 1.64b	71.38 ± 0.64ab	30.69 ± 0.64ab	31.51 ± 0.47a	9.61 ± 0.14a	7.53 ± 0.23a
	N3	686.67 ± 15.28a	340.00 ± 10.00a	95.96 ± 5.27ab	74.05 ± 2.57a	58.98 ± 1.57bc	66.74 ± 0.51cd	30.31 ± 0.45abc	30.97 ± 0.44a	9.48 ± 0.30ab	7.49 ± 0.57a
	Mean	665.56 ± 1.92A	320.00 ± 8.82A	88.47 ± 1.60B	67.28 ± 0.76AB	61.66 ± 0.27A	70.79 ± 0.94A	30.70 ± 0.32A	31.37 ± 0.37A	9.49 ± 0.15A	7.46 ± 0.19A
F-value	D	279.034**		18.295**		10.755**		2.515ns		42.889**	
	N	37.966**		73.696**		57.873**		5.578**		8.140**	
	Y	6008.379**		460.905**		442.705**		35.484**		670.136**	
	D × N	1.172ns		1.190ns		2.785*		0.215ns		1.313ns	

D1, D2 and D3 refer to the different planting density treatments (16.7 × 10^4^, 20.8 × 10^4^ and 27.8 × 10^4^ hills ha^-1^, respectively). N1, N2 and N3 refer to the different nitrogen fertilizer treatments (150, 225 and 300 kg ha^-1^, respectively). PN, SP, SR, GW and AY represent panicle number, spikelet number per panicle, seed setting rate, 1000-grain weight and actual yield, respectively. Different lowercase letters followed the values in the same column mean the significant difference of the different combined application of D and N levels at *p*< 0.05. Different uppercase letters mean the significant difference of different average D levels at *p*< 0.05. ANOVA *p* values and symbols were defined as: *p< 0.05; **p< 0.01; ns: *p* > 0.05, ns means non-significant. The data presented are the mean ± standard deviation, *n* = 3.

**Table 3:** Effects of planting density and nitrogen application rate combinations on ratoon rice yield of CKYSM.

Treatment		PN (×10^4^ ha^-1^)		SP		SR (%)		GW (g)		AY (t ha^-1^)	
		2022	2023	2022	2023	2022	2023	2022	2023	2022	2023
D1	N1	623.33 ± 23.09f	363.34 ± 5.77e	75.83 ± 2.75c	54.90 ± 4.12cd	68.69 ± 2.81abc	58.98 ± 2.6abc	23.45 ± 0.18a	25.58 ± 0.18a	8.05 ± 0.42b	5.69 ± 0.11e
	N2	673.34 ± 15.28e	376.67 ± 15.28de	82.32 ± 0.81a	59.49 ± 2.26b	66.68 ± 0.47cd	56.43 ± 2.49cd	23.09 ± 0.55a	25.43 ± 0.22a	8.35 ± 0.27ab	5.82 ± 0.05de
	N3	686.67 ± 25.17de	386.67 ± 5.77d	85.12 ± 2.98a	66.23 ± 2.84a	66.51 ± 1.71cd	54.00 ± 1.65d	22.88 ± 0.48a	25.29 ± 0.47a	8.57 ± 0.64ab	6.03 ± 0.18cd
	Mean	661.11 ± 5.09C	375.56 ± 5.09B	81.09 ± 1.84A	60.20 ± 2.37A	67.29 ± 1.56A	56.47 ± 1.81A	23.14 ± 0.24A	25.43 ± 0.21B	8.33 ± 0.29B	5.85 ± 0.07B
D2	N1	710.00 ± 10.00cd	433.33 ± 5.77c	70.73 ± 3.38d	52.81 ± 1.66cd	69.42 ± 1.28ab	61.04 ± 0.92ab	23.36 ± 0.14a	26.11 ± 0.16a	8.49 ± 0.44ab	6.03 ± 0.22cd
	N2	723.33 ± 20.81c	453.33 ± 11.55abc	76.75 ± 4.28bc	54.65 ± 4.02cd	66.97 ± 0.70bcd	58.71 ± 1.25abc	23.31 ± 0.44a	25.96 ± 0.22a	8.83 ± 0.10ab	6.12 ± 0.17bcd
	N3	740.00 ± 10.00bc	463.33 ± 5.77ab	83.99 ± 2.73a	57.00 ± 1.47bc	65.26 ± 0.79d	56.92 ± 1.48cd	22.65 ± 1.14a	25.85 ± 0.41a	8.91 ± 0.67ab	6.39 ± 0.03ab
	Mean	724.44 ± 7.70B	450.00 ± 5.77A	77.16 ± 0.98B	54.82 ± 1.48B	67.22 ± 0.83A	58.89 ± 0.84A	23.11 ± 0.35A	25.97 ± 0.25A	8.74 ± 0.16A	6.18 ± 0.09A
D3	N1	743.33 ± 5.78bc	446.67 ± 23.09bc	69.38 ± 0.69d	51.91 ± 1.60d	69.94 ± 1.17a	61.34 ± 2.19a	23.45 ± 0.26a	26.03 ± 0.55a	8.54 ± 0.34ab	6.15 ± 0.11bc
	N2	760.00 ± 20.00ab	463.33 ± 15.28ab	71.05 ± 1.64d	53.58 ± 1.66cd	68.42 ± 1.67abc	59.22 ± 1.25abc	23.35 ± 0.41a	25.99 ± 0.72a	9.00 ± 0.38a	6.49 ± 0.30a
	N3	783.33 ± 20.82a	476.67 ± 15.28a	80.76 ± 0.99ab	53.78 ± 2.29cd	65.21 ± 2.38d	58.03 ± 1.10bc	23.15 ± 0.33a	25.84 ± 0.67a	8.61 ± 0.47ab	6.32 ± 0.18abc
	Mean	762.22 ± 1.92A	462.22 ± 17.11A	73.73 ± 0.61C	53.09 ± 0.77B	67.86 ± 0.35A	59.53 ± 1.19A	23.31 ± 0.24A	25.95 ± 0.22A	8.72 ± 0.10A	6.32 ± 0.17A
F-value	D	161.981**		39.363**		6.170**		2.292ns		8.969**	
	N	22.633**		53.817**		28.015**		2.728ns		4.838*	
	Y	4222.562**		994.140**		459.167**		361.761**		754.779**	
	D × N	0.504ns		1.174ns		0.154ns		0.155ns		1.034ns	

D1, D2 and D3 refer to the different planting density treatments (16.7 × 10^4^, 20.8 × 10^4^ and 27.8 × 10^4^ hills ha^-1^, respectively). N1, N2 and N3 refer to the different nitrogen fertilizer treatments (150, 225 and 300 kg ha^-1^, respectively). PN, SP, SR, GW and AY represent panicle number, spikelet number per panicle, seed setting rate, 1000-grain weight and actual yield, respectively. Different lowercase letters followed the values in the same column mean the significant difference of the different combined application of D and N levels at *p*< 0.05. Different uppercase letters mean the significant difference of different average D levels at *p*< 0.05. ANOVA *p* values and symbols were defined as: *p< 0.05; **p< 0.01; ns: *p* > 0.05, ns means non-significant. The data presented are the mean ± standard deviation, *n* = 3.

As planting density increased, PN in FY498 first increased and then decreased, following the trend of D2 > D3 > D1, whereas CKYSM showed a continuous decline. The trends for SR and GW in response to planting density were inconsistent in both cultivars. In contrast, increasing nitrogen application led to a consistent increase in PN, while SR and GW showed a declining trend across both cultivars.

Across the two growing seasons, both cultivars exhibited the highest AY under either D2N3 or D3N2. Compared to the lowest treatment (D1N1), FY498 showed yield increases of 11.10% in 2022 and 30.24% in 2023, while CKYSM increased by 11.80% and 14.06%, respectively.

**Figure 4:**
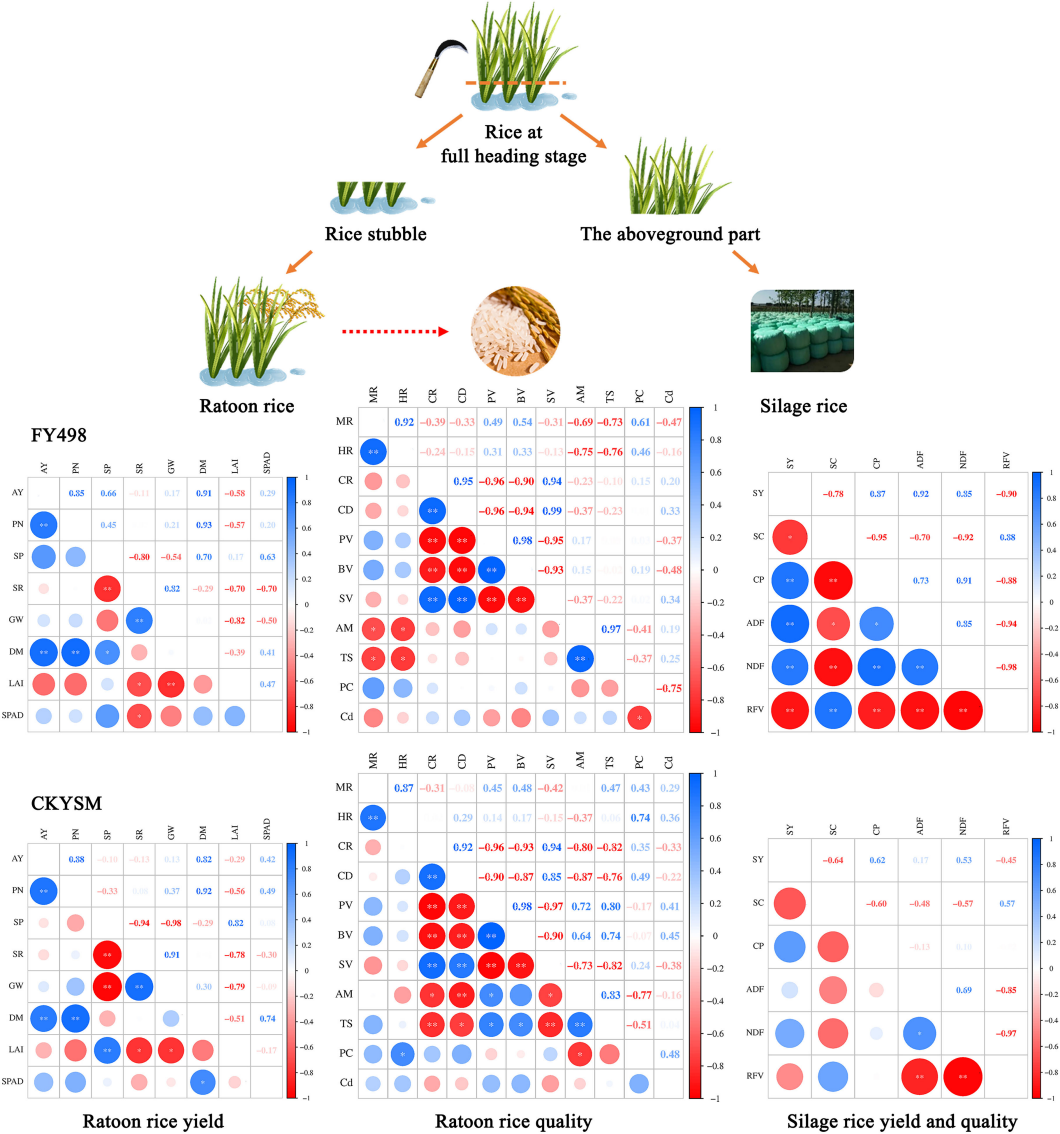
Correlation coefficients among silage rice yield and quality, ratoon rice yield and yield components, and grain quality traits. AY, PN, SP, SR, GW, DM, LAI, MR, HR, CR, CD, PV, BV, SV, AM, TS, PC, SY, SC, CP, ADF, NDF and RFV represent actual yield, panicle number, spikelet number per panicle, seed setting rate, 1000-grain weight, dry matter accumulation, leaf area index, milled rice rate, head milled rice rate, chalkiness rate, chalkiness degree, peak viscosity, breakdown viscosity, setback viscosity, amylose content, total starch content, protein content, silage rice yield, starch content, crude protein content, acid detergent fiber, neutral detergent fiber and relative feed value, respectively. ANOVA *p* values and symbols were defined as: *p< 0.05; **p< 0.01; ns: *p* > 0.05.

ANOVA results showed that planting density and nitrogen application had highly significant effects on AY and most yield components, except for SR. Their interaction effect was only significant for the SR in FY498. Correlation analysis revealed a highly significant positive correlation between the PN and AY, indicating that PN was the most critical factor contributing to yield improvement in ratoon rice (**Figure 4**).

### 3.6 Ratoon rice quality

#### 3.6.1 Milling and appearance quality

Moderate increases in nitrogen application during the first season improved the milling quality of ratoon rice, while higher planting density negatively affected it. Moreover, both increased planting density and nitrogen input led to deterioration in ratoon rice appearance quality (**Tables 4**, **5**).

**Table 4:** Effects of planting density and nitrogen application rate combinations on ratoon rice milling and appearance quality of FY498.

Treatment		BR (%)		MR (%)		HR (%)		CR (%)		CD (%)	
		2022	2023	2022	2023	2022	2023	2022	2023	2022	2023
D1	N1	81.28 ± 0.25a	77.33 ± 0.48a	69.19 ± 1.52a	68.15 ± 0.09abc	56.65 ± 0.19abc	57.24 ± 1.43ab	11.80 ± 0.52f	17.07 ± 0.38d	2.50 ± 0.17e	4.20 ± 0.26e
	N2	81.11 ± 0.32a	77.05 ± 1.05a	69.38 ± 1.05a	68.32 ± 1.29ab	57.08 ± 1.14ab	57.88 ± 1.05a	13.20 ± 0.78def	22.20 ± 1.91bc	3.87 ± 0.25d	5.40 ± 0.17d
	N3	80.41 ± 1.27a	77.36 ± 0.74a	69.27 ± 0.82a	69.34 ± 0.49a	57.68 ± 1.77a	58.07 ± 0.59a	14.63 ± 1.00cd	23.50 ± 1.35bc	4.73 ± 0.21bc	6.17 ± 0.21abc
	Mean	80.93 ± 0.37A	77.25 ± 0.29A	69.28 ± 0.80A	68.60 ± 0.48A	57.14 ± 0.85A	57.73 ± 0.41A	13.21 ± 0.52B	20.92 ± 0.44B	3.70 ± 0.17C	5.26 ± 0.11B
D2	N1	80.20 ± 0.98a	77.46 ± 1.32a	68.20 ± 0.90a	65.62 ± 2.14bc	54.26 ± 1.12cd	55.48 ± 1.81ab	12.27 ± 0.87ef	20.73 ± 0.38c	3.67 ± 0.15d	5.53 ± 0.31d
	N2	80.30 ± 1.57a	77.64 ± 1.13a	68.83 ± 1.52a	66.05 ± 0.95bc	56.93 ± 2.16abc	55.68 ± 0.57ab	13.50 ± 1.67def	22.70 ± 1.71bc	4.07 ± 0.29d	6.03 ± 0.12bc
	N3	79.82 ± 1.64a	76.88 ± 0.42a	68.66 ± 0.44a	66.20 ± 2.6bc	57.37 ± 1.48a	55.74 ± 1.37ab	16.93 ± 0.84ab	24.03 ± 1.62b	5.07 ± 0.31ab	6.37 ± 0.29ab
	Mean	80.11 ± 1.36A	77.33 ± 0.44A	68.56 ± 0.77B	65.96 ± 1.72AB	56.19 ± 0.74AB	55.64 ± 0.71A	14.23 ± 0.54B	22.49 ± 1.22B	4.27 ± 0.13B	5.98 ± 0.20A
D3	N1	80.59 ± 1.11a	77.35 ± 0.54a	67.96 ± 0.39a	65.35 ± 1.39c	53.64 ± 1.49d	54.88 ± 1.97b	13.77 ± 0.74de	23.20 ± 1.51bc	3.77 ± 0.29d	5.8 ± 0.36cd
	N2	80.96 ± 0.79a	76.94 ± 0.72a	68.29 ± 2.04a	65.94 ± 1.81bc	56.33 ± 1.42abcd	55.64 ± 1.23ab	15.57 ± 0.85bc	24.77 ± 1.86ab	4.53 ± 0.15c	6.20 ± 0.46abc
	N3	80.71 ± 0.95a	76.96 ± 1.26a	68.14 ± 1.75a	65.54 ± 0.54bc	54.34 ± 0.65bcd	55.34 ± 2.03ab	18.40 ± 0.92a	27.03 ± 1.33a	5.37 ± 0.31a	6.67 ± 0.12a
	Mean	80.75 ± 0.16A	77.08 ± 0.65A	68.13 ± 0.79C	65.61 ± 0.79B	54.77 ± 0.79B	55.29 ± 1.71A	15.91 ± 0.70A	25.00 ± 0.41A	4.56 ± 0.08A	6.22 ± 0.11A
F-value	D	0.704ns		13.195**		12.629**		32.085**		65.049**	
	N	0.722ns		0.647ns		3.825*		50.329**		163.163**	
	Y	172.104**		30.630**		0.225ns		573.963**		601.254**	
	D × N	0.158ns		0.139ns		0.677ns		0.853ns		8.192**	

D1, D2 and D3 refer to the different planting density treatments (16.7 × 10^4^, 20.8 × 10^4^ and 27.8 × 10^4^ hills ha^-1^, respectively). N1, N2 and N3 refer to the different nitrogen fertilizer treatments (150, 225 and 300 kg ha^-1^, respectively). BR, MR, HR, CR and CD represent brown rice rate, milled rice rate, head milled rice rate, chalkiness rate and chalkiness degree, respectively. Different lowercase letters followed the values in the same column mean the significant difference of the different combined application of D and N levels at *p*< 0.05. Different uppercase letters mean the significant difference of different average D levels at *p*< 0.05. ANOVA *p* values and symbols were defined as: * *p*< 0.05; ** *p*< 0.01; ns: *p* > 0.05, ns means non-significant. The data presented are the mean ± standard deviation, *n* = 3.

**Table 5:** Effects of planting density and nitrogen application rate combinations on ratoon rice milling and appearance quality of CKYSM.

Treatment		BR (%)		MR (%)		HR (%)		CR (%)		CD (%)	
		2022	2023	2022	2023	2022	2023	2022	2023	2022	2023
D1	N1	78.20 ± 1.05abc	74.20 ± 1.01ab	68.22 ± 1.50a	65.24 ± 0.82ab	51.22 ± 1.95bc	54.82 ± 0.4ab	1.53 ± 0.12e	6.87 ± 0.21c	0.30 ± 0.10d	2.10 ± 0.20f
	N2	78.37 ± 0.29ab	74.20 ± 0.81ab	68.34 ± 1.34a	65.28 ± 0.8ab	53.36 ± 1.35ab	54.97 ± 1.66ab	1.57 ± 0.12e	7.43 ± 0.49bc	0.37 ± 0.12d	2.50 ± 0.20cde
	N3	78.18 ± 1.01abc	74.83 ± 1.14ab	68.87 ± 0.59a	65.87 ± 1.22a	56.45 ± 1.78a	55.39 ± 1.08a	2.10 ± 0.10c	8.00 ± 0.40b	0.57 ± 0.06c	2.57 ± 0.21bcd
	Mean	78.25 ± 0.64A	74.41 ± 0.27A	68.47 ± 0.66A	65.46 ± 0.40A	53.68 ± 0.79A	55.06 ± 0.54A	1.73 ± 0.03C	7.43 ± 0.13B	0.41 ± 0.05B	2.39 ± 0.07B
D2	N1	77.38 ± 0.36bc	75.62 ± 0.61a	67.20 ± 0.17ab	64.46 ± 1.44ab	40.46 ± 1.51d	53.12 ± 1.47ab	1.57 ± 0.06e	6.97 ± 0.60c	0.37 ± 0.06d	2.20 ± 0.20ef
	N2	78.04 ± 0.3abc	75.29 ± 1.04a	67.40 ± 0.91ab	65.01 ± 0.59ab	50.94 ± 1.34bc	54.34 ± 1.44ab	1.93 ± 0.15cd	7.90 ± 0.53b	0.40 ± 0.10d	2.67 ± 0.15bcd
	N3	78.55 ± 0.38a	73.89 ± 1.8ab	68.22 ± 0.08a	65.48 ± 0.20ab	51.88 ± 3.83bc	55.15 ± 1.07ab	2.50 ± 0.20b	9.03 ± 0.38a	0.73 ± 0.06ab	2.83 ± 0.15abc
	Mean	77.99 ± 0.32A	74.93 ± 0.86A	67.61 ± 0.33B	64.98 ± 0.61A	47.76 ± 1.72B	54.20 ± 0.45AB	2.00 ± 0.12B	7.97 ± 0.40B	0.50 ± 0.00B	2.57 ± 0.15AB
D3	N1	77.83 ± 0.6abc	72.94 ± 0.89ab	65.72 ± 1.73b	63.20 ± 2.04b	35.92 ± 1.69e	52.56 ± 0.53b	1.77 ± 0.12de	8.80 ± 0.36a	0.37 ± 0.06d	2.40 ± 0.17def
	N2	78.27 ± 0.26ab	72.27 ± 2.40b	67.32 ± 0.83ab	64.38 ± 2.34ab	48.52 ± 3.18c	53.64 ± 1.15ab	2.13 ± 0.06c	9.00 ± 0.56a	0.63 ± 0.06bc	2.87 ± 0.21ab
	N3	77.08 ± 0.24c	72.76 ± 2.39ab	67.00 ± 1.59ab	63.53 ± 0.63ab	48.39 ± 3.89c	54.71 ± 2.05ab	2.77 ± 0.21a	9.10 ± 0.70a	0.83 ± 0.06a	3.07 ± 0.06a
	Mean	77.73 ± 0.29A	72.66 ± 0.84B	66.68 ± 0.93C	63.70 ± 0.69B	44.28 ± 2.47B	53.64 ± 0.47B	2.22 ± 0.08A	8.97 ± 0.32A	0.61 ± 0.04A	2.78 ± 0.07A
F-value	D	6.737**		10.608**		33.125**		41.066**		18.495**	
	N	0.140ns		2.406ns		40.027**		40.010**		49.112**	
	Y	165.849**		81.426**		108.951**		4473.563**		2735.063**	
D × N	0.239ns		0.718ns		3.792*		2.639ns		0.902ns	

D1, D2 and D3 refer to the different planting density treatments (16.7 × 10^4^, 20.8 × 10^4^ and 27.8 × 10^4^ hills ha^-1^, respectively). N1, N2 and N3 refer to the different nitrogen fertilizer treatments (150, 225 and 300 kg ha^-1^, respectively). BR, MR, HR, CR and CD represent brown rice rate, milled rice rate, head milled rice rate, chalkiness rate and chalkiness degree, respectively. Different lowercase letters followed the values in the same column mean the significant difference of the different combined application of D and N levels at *p*< 0.05. Different uppercase letters mean the significant difference of different average D levels at *p*< 0.05. ANOVA *p* values and symbols were defined as: * *p*< 0.05; ** *p*< 0.01; ns: *p* > 0.05, ns means non-significant. The data presented are the mean ± standard deviation, *n* = 3.

With increasing planting density and nitrogen input, BR showed no consistent pattern, and differences among most treatments were not statistically significant. MR and HR tended to decrease with increasing planting density. In contrast, nitrogen application promoted an increase or exhibited a parabolic trend in MR and HR (N2 > N3 > N1, D2 > D3 > D1). CR and CD both increased progressively with higher planting density and nitrogen input.

Across the two growing seasons, both FY498 and CKYSM achieved the highest MR and HR under D1N2 or D1N3 and the lowest under D3N1. Conversely, CR and CD were lowest under D1N1 and highest under D3N3. Compared to D3N1, the highest MR and HR of FY498 increased by 1.42 and 4.04 percentage points in 2022, and by 3.99 and 3.19 percentage points in 2023, respectively. For CKYSM, the corresponding increases were 3.15 and 20.53 percentage points in 2022, and 2.67 and 2.83 percentage points in 2023. Relative to D3N3, the lowest CR and CD of FY498 decreased by 6.60 and 2.87 percentage points in 2022, and by 9.96 and 2.47 percentage points in 2023; for CKYSM, CR and CD decreased by 1.24 and 0.53 percentage points in 2022, and by 2.23 and 0.97 percentage points in 2023.

ANOVA results showed that both planting density and nitrogen application had highly significant effects on all appearance quality parameters. Planting density also had a highly significant effect on MR and HR, while nitrogen application significantly or highly significantly affected HR but not MR.

#### 3.6.2 Nutritional and safety quality

Planting density and nitrogen application in the first season significantly affected both the nutritional and safety quality of ratoon rice, with cultivar-specific responses observed between FY498 and CKYSM (**Tables 6**, **7**).

**Table 6:** Effects of planting density and nitrogen application rate combinations on ratoon rice nutritional and safety quality of FY498.

Treatment		AC (g 100g^-1^)		TS (g 100g^-1^)		PC (%)		Cd (mg kg^-1^)	
		2022	2023	2022	2023	2022	2023	2022	2023
D1	N1	24.55 ± 0.31bc	16.61 ± 0.55abc	77.58 ± 3.90bc	58.43 ± 1.37ab	8.57 ± 0.07abc	7.39 ± 0.15a	0.0296 ± 0.0017e	0.0215 ± 0.0011f
	N2	23.13 ± 0.98cd	15.45 ± 0.38d	73.46 ± 2.67c	57.60 ± 1.17abc	8.60 ± 0.19ab	7.42 ± 0.19a	0.0357 ± 0.0016c	0.0273 ± 0.0016cd
	N3	22.30 ± 0.48d	11.63 ± 0.45e	71.90 ± 5.29c	52.76 ± 3.51c	8.68 ± 0.14a	7.63 ± 0.19a	0.0315 ± 0.0012de	0.0231 ± 0.001ef
	Mean	23.33 ± 0.31C	14.56 ± 0.29B	74.31 ± 3.37B	56.26 ± 1.04C	8.61 ± 0.05A	7.48 ± 0.15A	0.0323 ± 0.0013C	0.0240 ± 0.0007C
D2	N1	24.60 ± 0.71bc	16.92 ± 0.72ab	78.20 ± 3.24bc	59.48 ± 3.26ab	8.25 ± 0.15d	6.58 ± 0.15c	0.0378 ± 0.0024bc	0.0293 ± 0.0018bc
	N2	23.79 ± 0.84bcd	16.00 ± 0.40bcd	76.62 ± 1.53bc	59.18 ± 4.00ab	8.27 ± 0.23cd	6.60 ± 0.14c	0.0425 ± 0.0031a	0.0325 ± 0.0013a
	N3	22.89 ± 1.06d	15.58 ± 0.82cd	75.40 ± 1.90c	55.69 ± 4.22bc	8.37 ± 0.09bcd	6.95 ± 0.19b	0.0385 ± 0.0021bc	0.0303 ± 0.0016ab
	Mean	23.76 ± 0.46B	16.17 ± 0.62A	76.74 ± 1.94AB	58.12 ± 1.42B	8.30 ± 0.05B	6.71 ± 0.07B	0.0396 ± 0.0011A	0.0307 ± 0.0008A
D3	N1	27.42 ± 1.04a	17.43 ± 0.83a	84.70 ± 4.26a	61.81 ± 1.21a	8.26 ± 0.25cd	7.33 ± 0.09a	0.0350 ± 0.0026cd	0.0249 ± 0.0011de
	N2	25.24 ± 0.87b	16.50 ± 0.71abcd	82.61 ± 2.95ab	59.45 ± 2.83ab	8.34 ± 0.05bcd	7.35 ± 0.24a	0.0410 ± 0.0013ab	0.0305 ± 0.0024ab
	N3	23.59 ± 0.84cd	15.61 ± 0.78cd	75.83 ± 1.52c	58.37 ± 2.78ab	8.38 ± 0.15bcd	7.56 ± 0.25a	0.0314 ± 0.0007de	0.0259 ± 0.0013de
	Mean	25.42 ± 0.12A	16.51 ± 0.48A	81.05 ± 1.79A	59.88 ± 1.58A	8.33 ± 0.10B	7.42 ± 0.17A	0.0358 ± 0.0009B	0.0271 ± 0.0015B
F-value	D	33.857**		13.110**		44.111**		68.164**	
	N	58.551**		12.591**		6.407**		46.615**	
	Y	1763.629**		540.742**		629.857**		307.043**	
	D × N	3.561*		0.391ns		0.162ns		2.495ns	

D1, D2 and D3 refer to the different planting density treatments (16.7 × 10^4^, 20.8 × 10^4^ and 27.8 × 10^4^ hills ha^-1^, respectively). N1, N2 and N3 refer to the different nitrogen fertilizer treatments (150, 225 and 300 kg ha^-1^, respectively). AC, TS and PC represent amylose content, total starch content and protein content, respectively. Different lowercase letters followed the values in the same column mean the significant difference of the different combined application of D and N levels at *p*< 0.05. Different uppercase letters mean the significant difference of different average D levels at *p*< 0.05. ANOVA *p* values and symbols were defined as: *p< 0.05; **p< 0.01; ns: *p* > 0.05, ns means non-significant. The data presented are the mean ± standard deviation, *n* = 3.

**Table 7:** Effects of planting density and nitrogen application rate combinations on ratoon rice nutritional and safety quality of CKYSM.

Treatment		AC (g 100g^-1^)		TS (g 100g^-1^)		PC (%)		Cd (mg kg^-1^)	
		2022	2023	2022	2023	2022	2023	2022	2023
D1	N1	22.30 ± 0.48a	18.85 ± 0.91ab	69.89 ± 1.78ab	80.14 ± 3.48a	8.92 ± 0.09bc	8.16 ± 0.15ab	0.0518 ± 0.0036ab	0.0438 ± 0.0034a
	N2	20.23 ± 0.50c	17.48 ± 0.82bc	66.29 ± 3.48bcd	78.62 ± 0.92a	9.18 ± 0.18ab	8.25 ± 0.16ab	0.0505 ± 0.003ab	0.0446 ± 0.0023a
	N3	20.21 ± 0.84c	17.03 ± 0.41c	60.61 ± 3.78d	76.17 ± 4.74ab	9.29 ± 0.18a	8.38 ± 0.22a	0.0502 ± 0.0021ab	0.0409 ± 0.0018b
	Mean	20.91 ± 0.55A	17.78 ± 0.19A	65.60 ± 2.14A	78.31 ± 0.42A	9.13 ± 0.14A	8.26 ± 0.12A	0.0508 ± 0.0009A	0.0431 ± 0.0025A
D2	N1	22.81 ± 0.27a	19.16 ± 1.01a	72.52 ± 3.20a	80.29 ± 3.84a	8.72 ± 0.23c	7.68 ± 0.12c	0.0438 ± 0.0022cd	0.0349 ± 0.0024d
	N2	21.82 ± 0.40a	17.98 ± 0.97abc	66.94 ± 4.89abc	79.90 ± 1.59a	8.86 ± 0.10bc	8.07 ± 0.14ab	0.0474 ± 0.0029bc	0.0382 ± 0.0018c
	N3	20.53 ± 0.53bc	17.21 ± 1.02bc	64.39 ± 2.45bcd	77.96 ± 2.80a	8.93 ± 0.11bc	8.13 ± 0.22ab	0.0394 ± 0.0020d	0.0335 ± 0.0007d
	Mean	21.72 ± 0.13A	18.12 ± 0.45A	67.95 ± 0.98A	79.39 ± 1.96A	8.84 ± 0.03A	7.96 ± 0.07B	0.0435 ± 0.0011B	0.0355 ± 0.0013C
D3	N1	21.62 ± 0.88ab	17.66 ± 0.88abc	62.60 ± 4.14cd	70.27 ± 0.20bc	8.86 ± 0.23bc	7.98 ± 0.18bc	0.0463 ± 0.0041bc	0.0355 ± 0.0015d
	N2	18.88 ± 0.72d	17.07 ± 0.89c	62.33 ± 2.43cd	70.14 ± 5.88bc	9.08 ± 0.22ab	8.17 ± 0.20ab	0.0541 ± 0.0014a	0.0455 ± 0.0016a
	N3	18.72 ± 0.91d	16.53 ± 0.47c	51.97 ± 1.86e	66.41 ± 2.11c	9.20 ± 0.07ab	8.22 ± 0.14ab	0.0462 ± 0.0038bc	0.0348 ± 0.0021d
	Mean	19.74 ± 0.39B	17.09 ± 0.5A	58.97 ± 1.62B	68.94 ± 1.20B	9.05 ± 0.13A	8.12 ± 0.08AB	0.0489 ± 0.0025A	0.0386 ± 0.0013B
F-value	D	16.995**		44.641**		13.078**		43.050**	
	N	32.307**		17.724**		14.154**		28.141**	
	Y	215.030**		161.124**		339.188**		173.383**	
	D × N	0.515ns		0.849ns		0.114ns		6.214**	

D1, D2 and D3 refer to the different planting density treatments (16.7 × 10^4^, 20.8 × 10^4^ and 27.8 × 10^4^ hills ha^-1^, respectively). N1, N2 and N3 refer to the different nitrogen fertilizer treatments (150, 225 and 300 kg ha^-1^, respectively). AC, TS and PC represent amylose content, total starch content and protein content, respectively. Different lowercase letters followed the values in the same column mean the significant difference of the different combined application of D and N levels at *p*< 0.05. Different uppercase letters mean the significant difference of different average D levels at *p*< 0.05. ANOVA *p* values and symbols were defined as: **p< 0.01; ns: *p* > 0.05, ns means non-significant. The data presented are the mean ± standard deviation, *n* = 3.

With increasing planting density, FY498 exhibited a gradual increase in both AC and TS contents, while Cd content followed a non-linear trend, first increasing and then decreasing (D2 > D3 > D1). In contrast, CKYSM showed a peak in AC and TS contents at medium density (D2 > D1 > D3), while Cd content first decreased and then increased. Protein content in both cultivars followed a similar trend, first decreasing and then increasing with increasing density (D1 > D3 > D2). These results indicated that higher planting density favored starch accumulation in FY498 but might compromise protein content and increase Cd accumulation, thereby hindering the coordinated improvement of nutritional and safety quality. Conversely, moderate planting density in CKYSM promoted starch accumulation while reducing Cd content, contributing to a more favorable balance between nutritional and safety traits.

As nitrogen input increased, both cultivars showed a gradual decline in AC and TS contents, while protein content increased steadily. Cd content increased first and then decreased with nitrogen input. These findings indicated that nitrogen application tended to enhance protein synthesis at the expense of carbohydrate accumulation and might simultaneously increase the risk of Cd accumulation in rice grains.

Across the two growing seasons, FY498 achieved the highest AC and TS contents under D3N1, while CKYSM reached its maximum under D2N1. For both cultivars, the highest protein content was observed under D1N3. Regarding Cd content, FY498 had the lowest levels under D1N1 and the highest under D2N2, whereas CKYSM exhibited the lowest Cd content under D2N3 and the highest under D3N2.

In summary, for FY498, a combination of low planting density and high nitrogen application (D1N3) achieved a relatively optimal balance between nutritional and safety quality. For CKYSM, the combination of moderate planting density and high nitrogen input (D2N3) was more conducive to the simultaneous improvement of both quality dimensions.

#### 3.6.3 RVA profile characters

RVA profile characteristics are closely associated with rice cooking and eating quality. Generally, higher PV and BV, along with lower SV, correspond to better eating quality. Both planting density and nitrogen application in the first season significantly affected PV, BV, and SV, with increased planting density and nitrogen input leading to reductions in RVA parameters (**Tables 8**, **9**). The complete data of RVA profile characters is available in Supplementary **Supplementary Table S4, S5**.

**Table 8:** Effects of planting density and nitrogen application rate combinations on ratoon rice RVA profile characters of FY498.

Treatment		PV (RVU)		BV (RVU)		SV (RVU)		PeT (min)		PaT (°C)	
		2022	2023	2022	2023	2022	2023	2022	2023	2022	2023
D1	N1	249.92 ± 4.91a	314.00 ± 12.14a	92.50 ± 1.52a	125.56 ± 5.50a	39.53 ± 4.20a	41.72 ± 2.00f	6.07 ± 0.07a	6.02 ± 0.14a	77.40 ± 0.43a	81.83 ± 1.93b
	N2	246.60 ± 7.75a	283.17 ± 3.19b	89.93 ± 4.71a	111.86 ± 8.99b	41.10 ± 4.43a	65.19 ± 5.46de	6.00 ± 0.13a	5.62 ± 0.17b	76.87 ± 0.03a	80.70 ± 0.85bcd
	N3	244.92 ± 7.17ab	251.64 ± 7.72ef	89.56 ± 7.00a	89.67 ± 7.87cd	42.30 ± 7.37a	77.08 ± 5.49abc	6.05 ± 0.20a	5.98 ± 0.25a	77.27 ± 0.46a	79.10 ± 0.80cd
	Mean	247.14 ± 3.01A	282.94 ± 5.18A	90.66 ± 1.41A	109.03 ± 3.25A	40.98 ± 2.37A	61.33 ± 4.11B	6.04 ± 0.07A	5.87 ± 0.11A	77.18 ± 0.26A	80.54 ± 1.09B
D2	N1	245.69 ± 1.90a	276.86 ± 3.14bc	89.47 ± 4.75a	99.31 ± 7.89c	41.65 ± 4.33a	60.86 ± 4.79e	6.11 ± 0.17a	5.91 ± 0.03a	77.45 ± 0.44a	78.65 ± 1.78d
	N2	241.11 ± 8.17abc	259.22 ± 8.25de	85.00 ± 7.72a	90.50 ± 3.91cd	43.28 ± 2.40a	68.83 ± 6.46bcde	6.08 ± 0.02a	5.93 ± 0.07a	76.92 ± 0.08a	76.30 ± 1.20e
	N3	240.11 ± 1.45abc	244.58 ± 5.90fg	88.11 ± 6.32a	83.20 ± 5.75de	43.59 ± 6.45a	80.03 ± 7.76ab	6.13 ± 0.07a	5.89 ± 0.03ab	77.17 ± 0.38a	79.65 ± 0.61bcd
	Mean	242.30 ± 2.13A	260.22 ± 3.63B	87.53 ± 2.01A	91.00 ± 4.67B	42.84 ± 2.16A	69.90 ± 2.34AB	6.11 ± 0.08A	5.91 ± 0.04A	77.18 ± 0.25A	78.20 ± 0.45C
D3	N1	239.53 ± 9.34abc	264.89 ± 16.77cd	88.47 ± 4.31a	96.17 ± 7.21c	41.78 ± 8.84a	67.86 ± 6.4cde	6.00 ± 0.07a	6.00 ± 0.07a	76.95 ± 0.00a	79.92 ± 1.40bcd
	N2	233.75 ± 1.18bc	242.31 ± 2.31fg	87.53 ± 1.83a	80.25 ± 6.82de	44.13 ± 4.13a	72.80 ± 10.67abcd	6.18 ± 0.14a	5.98 ± 0.10a	77.40 ± 0.87a	81.03 ± 0.75bc
	N3	230.16 ± 4.45c	233.36 ± 6.30g	87.19 ± 3.34a	76.16 ± 2.01e	45.42 ± 2.57a	81.11 ± 0.55a	6.13 ± 0.07a	5.78 ± 0.25ab	77.13 ± 0.45a	85.10 ± 0.96a
	Mean	234.48 ± 3.47B	246.85 ± 7.02C	87.73 ± 1.74A	84.19 ± 4.79B	43.78 ± 4.05A	73.93 ± 4.37A	6.10 ± 0.03A	5.92 ± 0.06A	77.16 ± 0.44A	82.02 ± 0.29A
F-value	D	60.868**		24.789**		8.041**		0.932ns		21.092**	
	N	60.946**		19.954**		21.051**		0.651ns		6.130**	
	Y	148.312**		13.155**		261.301**		23.049**		163.456**	
	D × N	2.395ns		1.602ns		1.611ns		2.795*		9.673**	

D1, D2 and D3 refer to the different planting density treatments (16.7 × 10^4^, 20.8 × 10^4^ and 27.8 × 10^4^ hills ha^-1^, respectively). N1, N2 and N3 refer to the different nitrogen fertilizer treatments (150, 225 and 300 kg ha^-1^, respectively). PV, BV, SV, PeT and PaT represent peak viscosity, breakdown viscosity, setback viscosity, peak time and pasting temperature, respectively. Different lowercase letters followed the values in the same column mean the significant difference of the different combined application of D and N levels at *p*< 0.05. Different uppercase letters mean the significant difference of different average D levels at *p*< 0.05. ANOVA *p* values and symbols were defined as: *p< 0.05; **p< 0.01; ns: *p* > 0.05, ns means non-significant. The data presented are the mean ± standard deviation, *n* = 3.

**Table 9:** Effects of planting density and nitrogen application rate combinations on ratoon rice RVA profile characters of CKYSM.

Treatment		PV (RVU)		BV (RVU)		SV (RVU)		PeT (min)		PaT (°C)	
		2022	2023	2022	2023	2022	2023	2022	2023	2022	2023
D1	N1	211.03 ± 2.59a	223.11 ± 10.88a	87.27 ± 5.99a	86.67 ± 3.91a	40.25 ± 7.47c	66.86 ± 8.01c	6.09 ± 0.10ab	4.49 ± 0.36b	84.13 ± 1.22a	84.35 ± 1.26bc
	N2	203.86 ± 1.67b	214.75 ± 8.07ab	86.47 ± 0.95a	81.97 ± 3.61ab	42.28 ± 3.03bc	71.55 ± 4.00bc	5.95 ± 0.04cd	5.80 ± 0.23a	81.98 ± 0.51b	81.25 ± 1.17d
	N3	199.08 ± 3.43c	201.33 ± 3.04bcd	84.36 ± 1.33a	78.83 ± 5.51bc	43.97 ± 0.91abc	81.25 ± 0.15ab	5.98 ± 0.10bcd	5.87 ± 0.07a	81.25 ± 0.44bc	86.68 ± 1.11a
	Mean	204.66 ± 1.51A	213.06 ± 0.94A	86.04 ± 1.77A	82.49 ± 4.16A	42.17 ± 1.79B	73.22 ± 1.37B	6.01 ± 0.08A	5.38 ± 0.10B	82.46 ± 0.52A	84.09 ± 0.48A
D2	N1	201.39 ± 1.79bc	208.42 ± 3.54bc	86.11 ± 2.84a	76.00 ± 3.46bcd	42.56 ± 3.84bc	74.44 ± 2.80abc	6.11 ± 0.03a	5.76 ± 0.21a	80.42 ± 0.45c	82.77 ± 0.28cd
	N2	192.33 ± 1.74d	204.19 ± 7.00bc	78.78 ± 1.85b	73.03 ± 4.1cd	44.25 ± 1.44abc	76.45 ± 2.46abc	5.91 ± 0.03d	5.87 ± 0.00a	77.55 ± 1.39e	85.92 ± 0.81ab
	N3	187.19 ± 2.72e	186.00 ± 9.29ef	77.50 ± 2.46b	63.50 ± 1.56e	44.42 ± 2.97abc	83.44 ± 3.42a	5.95 ± 0.04cd	5.75 ± 0.20a	80.45 ± 0.43c	84.23 ± 0.98bc
	Mean	193.64 ± 1.76B	199.54 ± 2.37B	80.79 ± 1.12B	70.84 ± 1.40B	43.74 ± 1.78B	78.11 ± 2.63AB	5.99 ± 0.01A	5.79 ± 0.01A	79.47 ± 0.56B	84.31 ± 0.68A
D3	N1	191.42 ± 2.53d	195.22 ± 5.29cde	77.86 ± 3.80b	72.83 ± 6.28cd	46.25 ± 0.6abc	80.98 ± 5.55ab	5.96 ± 0.08cd	5.78 ± 0.16a	80.22 ± 0.46cd	87.82 ± 0.80a
	N2	184.08 ± 1.40e	188.33 ± 6.60def	74.03 ± 3.09bc	68.67 ± 4.84de	47.61 ± 2.7ab	81.25 ± 1.40ab	6.11 ± 0.08a	5.69 ± 0.10a	78.88 ± 0.93de	78.95 ± 1.60e
	N3	178.53 ± 4.65f	178.75 ± 4.40f	70.08 ± 3.69c	62.19 ± 5.14e	49.30 ± 2.44a	84.28 ± 10.15a	6.04 ± 0.08abc	5.82 ± 0.04a	82.28 ± 0.78b	87.88 ± 0.55a
	Mean	184.68 ± 1.68C	187.43 ± 5.33C	73.99 ± 3.07C	67.90 ± 2.78B	47.72 ± 0.53A	82.17 ± 1.13A	6.04 ± 0.05A	5.76 ± 0.09A	80.46 ± 0.30B	84.88 ± 0.33A
F-value	D	81.903**		62.041**		11.480**		11.186**		9.680**	
	N	43.658**		23.995**		7.793**		11.028**		53.122**	
	Y	15.261**		43.677**		723.131**		83.153**		198.202**	
	D × N	0.277ns		0.842ns		0.642ns		12.111**		17.201**	

D1, D2 and D3 refer to the different planting density treatments (16.7 × 10^4^, 20.8 × 10^4^ and 27.8 × 10^4^ hills ha^-1^, respectively). N1, N2 and N3 refer to the different nitrogen fertilizer treatments (150, 225 and 300 kg ha^-1^, respectively). PV, BV, SV, PeT and PaT represent peak viscosity, breakdown viscosity, setback viscosity, peak time and pasting temperature, respectively. Different lowercase letters followed the values in the same column mean the significant difference of the different combined application of D and N levels at *p*< 0.05. Different uppercase letters mean the significant difference of different average D levels at *p*< 0.05. ANOVA *p* values and symbols were defined as: **p< 0.01; ns: *p* > 0.05, ns means non-significant. The data presented are the mean ± standard deviation, *n* = 3.

As planting density and nitrogen application increased, PV and BV exhibited a declining trend, while SV showed an increasing trend. Peak time and pasting temperature, however, did not show clear responses to changes in planting density or nitrogen levels.

Across the two growing seasons, the highest PV and BV and the lowest SV for both cultivars were observed under D1N1. Conversely, D3N3 resulted in the lowest PV and BV and the highest SV. Compared to D1N1, the D3N3 treatment reduced PV in FY498 and CKYSM by 7.91% and 15.40% in 2022, and 25.68% and 19.88% in 2023, respectively. BV decreased by 5.74% and 19.70% in 2022, and 39.34% and 28.25% in 2023 for FY498 and CKYSM, respectively. SV increased by 14.90% and 22.48% in 2022, and 94.44% and 26.05% in 2023 for the two cultivars, respectively.

### 3.7 Principal component analysis

A total of 13 indicators-including silage rice yield, relative feed value, ratoon rice yield, milled rice rate, head rice rate, chalkiness rate, chalkiness degree, peak viscosity, breakdown viscosity, setback viscosity, total starch content, protein content, and cadmium content-were subjected to principal component analysis (PCA) using SPSS. The data of eigen value, variance and cumulative variance is available in **Supplementary Tables S6**, **S7**.

The PCA results showed that D1N1 achieved the highest comprehensive score, characterized by superior silage rice and ratoon rice quality but lower yield (**Table 10**). Under D1N1, FY498 had low grain Cd content, while CKYSM showed elevated Cd levels. In contrast, D3N3 achieved the lowest comprehensive score, associated with high yield but poor quality and low Cd accumulation in both cultivars.

**Table 10:** The scores and rankings under different planting density and nitrogen application rate.

Treatment	FY498		CKYSM	
	Score	Ranking	Score	Ranking
D1N1	1.344	1	0.990	1
D1N2	0.646	2	0.839	2
D1N3	0.497	3	0.358	3
D2N1	-0.083	4	0.216	4
D2N2	-0.437	6	0.184	5
D2N3	-0.531	7	-0.292	6
D3N1	-0.235	5	-0.772	8
D3N2	-0.617	9	-0.493	7
D3N3	-0.584	8	-1.031	9

D1, D2 and D3 refer to the different planting density treatments (16.7 × 10^4^, 20.8 × 10^4^ and 27.8 × 10^4^ hills ha^-1^, respectively). N1, N2 and N3 refer to the different nitrogen fertilizer treatments (150, 225 and 300 kg ha^-1^, respectively).

Among the tested combinations, FY498 under D3N1 and CKYSM under D2N2 ranked fifth in overall performance. These treatments enabled the simultaneous achievement of relatively high first-season silage rice yield and second-season ratoon rice yield, while maintaining a favorable balance between silage rice quality and ratoon rice quality.

## 4 Discussion

### 4.1 Effects of planting density and nitrogen application rate in the first season on silage rice yield and quality

In recent years, with the rapid development of animal husbandry in China, the demand for feed has increased substantially, leading to growing pressure on feed resources. The conflicts of “land competition between food and feed” and “grain competition between humans and livestock” have become increasingly prominent, posing a serious constraint on the sustainable development of the livestock sector (Wu et al., 2023; Luo et al., 2005). Against this backdrop, the efficient utilization of crop residues-particularly as silage-has become a key strategy for recycling agricultural waste, reducing environmental pollution, and enhancing feed supply capacity (Chu et al., 2016). As the cropping structure evolves and herbivorous livestock sectors such as dairy farming expand, the importance of silage feed continues to rise (Cao et al., 2018; Chen et al., 2017). Dual-purpose ratoon rice, which enables forage production in the main season and grain production in the ratoon season by capitalizing on its regenerative capacity, offers a promising pathway to alleviate the feed shortage problem.

This study found that moderately increasing planting density and nitrogen application in the first season improved silage rice yield. This finding aligns with previous results in dual-purpose maize, where high planting density combined with high nitrogen input was shown to enhance forage yield (Li, 2020). However, as planting density and nitrogen input increased, the silage rice quality declined. According to the national grading standard for silage maize (GB/T 25882–2010), CP and starch contents in most treatments of both cultivars reached first- or second-grade standards, while ADF and NDF contents were more favorable under low to moderate planting density and nitrogen levels. These results indicated that higher yields may come at the expense of quality, which contrasts with previous conclusions drawn from dual-purpose maize research, where yield and quality improvements were found to be synergistic (Li, 2014).

This divergence might come from fundamental differences between the two crops. Silage maize is a C4 plant with high photosynthetic efficiency and strong adaptability at the population level. Moreover, many silage maize cultivars are specifically bred for whole-plant utilization, with the grain contributing significantly to overall feed quality (Chen, 2013). In contrast, rice is a C3 crop with relatively lower photosynthetic efficiency, and currently, no specialized forage rice cultivars have been developed in China (Hashida et al., 2018). Furthermore, at the time of harvest (after heading), the proportion of grain in the aboveground biomass is relatively low in rice, which weakens its positive contribution to overall forage quality and exacerbates the decline in nutritive value. Correlation analysis in this study also revealed a negative relationship between silage yield and quality in rice, underscoring the importance of balancing these traits through targeted cultivation practices (**Figure 4**). Thus, exploring optimized management strategies that can harmonize yield and quality is crucial for the efficient utilization of dual-purpose ratoon rice.

In summary, while the impact of cultivation practices on yield and quality in dual-purpose maize has been extensively studied (Ferreira and Teets, 2017; Ge et al., 2019), research on the forage use of rice straw remains limited. Existing studies primarily focus on factors such as cutting stage and stubble height, with relatively little attention paid to planting density and nitrogen input (Zhang et al., 2023b; Chen et al., 2022; Wu et al., 2024). Moreover, the underlying physiological and morphological reasons for the differential responses of rice and maize forage traits to planting density and nitrogen remain unclear and require further investigation. Currently, China lacks dedicated quality evaluation standards for rice used as forage, and researchers often rely on silage maize standards (GB/T 25882–2010) for assessment (Jian et al., 2024). Therefore, there is an urgent need to strengthen breeding programs for forage-specific rice cultivars, develop supporting cultivation technologies, and establish a comprehensive quality evaluation system to support the large-scale adoption of dual-purpose ratoon rice.

### 4.2 Effects of planting density and nitrogen application rate in the first season on ratoon rice yield and quality

Appropriate planting density and nitrogen application are key agronomic practices for achieving high rice yields. Previous studies have demonstrated that maximizing yield under different planting densities requires corresponding nitrogen levels. Specifically, under medium to low planting densities, early-season rice yield tends to increase with nitrogen application, whereas under high-density conditions, yield follows a trend of first increasing and then decreasing. In addition, nitrogen application in the early season shows limited impact on ratoon season yield (Luo, 2007). It has also been reported that early- and ratoon-season yields follow a parabolic relationship with nitrogen input, peaking at 253.9 kg ha^−1^ and 307.0 kg ha^−1^, respectively (Zheng et al., 2004). Furthermore, increasing planting density generally enhances both early- and ratoon-season yields, while nitrogen input showed the trend of first increasing and then decreasing. However, the effects of different density–nitrogen combinations on milling and appearance quality traits are relatively minor in hybrid mid-season rice (Jiang et al., 2019). This study findings further support these observations. In terms of yield, appropriately increasing planting density in the first season significantly enhanced PN and DM accumulation in the ratoon season, thereby contributing to higher ratoon rice yield. ANOVA revealed a significant interaction effect between planting density and nitrogen application rate on DM accumulation. DM accumulation and SPAD value were positively correlated with rice yield, with correlation coefficients of 0.91 and 0.29, respectively, while LAI showed a negative correlation with yield (correlation coefficient of -0.58). Under treatments with medium to high planting density and nitrogen levels, both DM accumulation and SPAD remained at relatively high levels during the grain-filling stage, indicating that high levels of these indicators have strong yield-enhancing potential. Efficient nutrient utilization and solar radiation interception after heading are crucial for ensuring rice yield (Hou et al., 2019). Notably, both cultivars achieved maximum yield under D2N3 or D3N2 demonstrating a strong yield enhancing effect consistent with previous reports (Wang, 2019).

However, these yield benefits were accompanied by declines in rice quality. While higher density and nitrogen input improved yield, they exerted adverse effects on milling, appearance, and nutritional quality. High-density (D3) and high-nitrogen (N3) treatments significantly reduced MR and HR, increased CR and CD, and worsened RVA profile characters (with decreased PV and BV, and increased SV), all of which suggested potential deterioration in eating quality. These trends mirror those observed in single-season rice (Chen et al., 2024). Regarding nutritional quality, nitrogen application elevated protein content but suppressed starch biosynthesis, leading to reductions in both amylose and total starch content. Moreover, increased nitrogen input was associated with elevated Cd content in the grain, which might pose food safety risks (≤0.2 mg kg^−1^) (Shi et al., 2023).

### 4.3 Exploring strategies for the synergistic improvement of silage rice and ratoon rice yield and quality

In this study, PCA based on 13 key indicators was conducted to systematically evaluate the effects of different planting density and nitrogen application combinations on the yield and quality of silage (first season) and grain (ratoon season) in dual-purpose ratoon rice. The analysis revealed that D1N1 achieved the highest comprehensive score for both cultivars, indicating superior forage and grain quality. However, this treatment was associated with relatively low yields, which might limit overall system profitability. In contrast, D3N3, despite producing high forage and grain yields and exhibiting low Cd accumulation in grain, showed inferior quality traits, resulting in the lowest overall score. These findings highlighted the inherent difficulty in achieving both high yield and high quality simultaneously.

The decline in quality observed under D3N3 may be primarily attributed to imbalances in assimilate allocation, carbon-nitrogen dynamics, and a reduction in grain-filling capacity. Studies have shown that increased nitrogen application promotes grain protein synthesis but suppresses starch accumulation, thereby disrupting the deposition structure of protein and starch in the endosperm, leading to increased chalkiness, a looser grain structure, and ultimately compromising both appearance and eating quality of the rice. Higher protein content has also been associated with reduced stickiness and elasticity of cooked rice, resulting in diminished taste value (Liu et al., 2024). In addition, high planting density and nitrogen levels alter the population structure of rice plants, reducing light penetration and ventilation of photosynthetic organs and causing source-sink imbalance. These changes result in insufficient grain filling and poor grain plumpness, which reduce the head milled rice rate. Simultaneously, the activities of key enzymes involved in starch biosynthesis are inhibited, further constraining starch accumulation and its structural integrity, ultimately exacerbating the deterioration of RVA profile characters (Li et al., 2018).

Notably, FY498 under D3N1 and CKYSM under D2N2 achieved relatively high comprehensive scores, suggesting that moderate-to-high planting densities combined with moderate nitrogen input are beneficial for balancing yield and quality, thereby supporting a synergistic forage-grain production model. For FY498, D3N1 ensured a high PN and substantial DM accumulation without significantly compromising LAI and SPAD value. MR and HR were above average, RVA profile characters were favorable, and Cd content remained at a relatively low level. For CKYSM, D2N2 exhibited better quality coordination, with low grain Cd content, a balanced profile of starch and protein contents, and good forage quality performance.

Taken together, these results suggest that optimizing the trade-off between planting density and nitrogen input was essential for dual-purpose ratoon rice production. Specifically, D3N1 is suitable for FY498 to achieve a coordinated improvement in yield, quality, and safety, while D2N2 is more conducive for CKYSM to attain dual excellence in both forage and grain quality. Future research could focus on the effects of other crop management practices, such as irrigation, weed and pest control, on the yield and quality of forage rice in the first season as well as the yield and quality of ratoon rice in the regeneration season.

## 5 Conclusion

A low planting density combined with low nitrogen input (D1N1) was favorable for achieving superior silage rice and better ratoon rice quality; however, yield performance under this treatment was relatively low. In contrast, the combination of high planting density and high nitrogen input (D3N3) resulted in high silage rice and ratoon rice yields as well as lower grain Cd accumulation. However, these combinations were associated with reductions in silage rice and ratoon rice quality. D3N1 was more suitable for FY498 and D2N2 was optimal for CKYSM to achieve relatively higher yield and quality in both silage season and ratoon season at the same time. These findings provide theoretical support for forage-grain dual-purpose ratoon rice cultivation and suggest that flexible cultivation management strategies can be adopted in practice depending on specific production goals.

## Data Availability

The original contributions presented in the study are included in the article/**Supplementary Material**. Further inquiries can be directed to the corresponding authors.

## References

[B1] Cao Z. H. Huang Y. L. Hao J. M. (2018). Multi-suitability comprehensive evaluation of crop straw resource utilization in China. Res. Environ. Sci. 31, 179–186. doi: 10.13198/j.issn.1001-6929.2017.03.58

[B2] Chen C. L. Yang Y. Hu L. Xie G. H. (2017). Review on the development of crop residue management policies in provincial regions in China. J. China Agric. Univ. 22, 1–16. doi: 10.11841/j.issn.1007-4333.2017.11.01

[B3] Chen G. Y. Li C. M. Hu M. M. He X. M. Yang H. Zhang Q. Q.. (2024). Evaluating rice lipid content, yield, and quality in response to nitrogen application rate and planting density. Front. Plant Sci. 15. doi: 10.3389/fpls.2024.1469264, PMID: 39619842 PMC11604421

[B4] Chen H. F. Yang D. Liang Y. Y. Zhang Z. X. Liang K. J. Lin W. X. (2010). Effect of nitrogen application strategy in the first cropping rice on dry matter accumulation, grain yield and nitrogen utilization efficiency of the first cropping rice and its ratoon rice crop. Chin. J. Eco-Agric. 18, 50–56. doi: 10.3724/SP.J.1011.2010.00050

[B5] Chen L. L. (2013). Effects of plant density and nitrogen rate on the silage yield and quality of foodstuff maize (Lanzhou, Gansu: Master Dissertation of Gansu Agricultural University).

[B6] Chen Y. W. Zheng H. B. Wang W. Q. Kuang N. Luo Y. Y. Zou D.. (2022). Effect of mowing treatment on the main season whole plant biomass and silage quality and yield in regeneration season of rationing rice. J. Agric. Sci. Technol. 24, 161–171. doi: 10.13304/j.nykjdb.2021.0117

[B7] Chen Z. Chen H. Jiang Y. Wang J. Khan A. Li P.. (2020). Metabolomic analysis reveals metabolites and pathways involved in grain quality traits of high-quality rice cultivars under a dry cultivation system. Food Chem. 326, 126845. doi: 10.1016/j.foodchem.2020.126845, PMID: 32438226

[B8] Chu T. S. Yang Z. L. Han L. J. (2016). Analysis on satisfied degree and advantage degree of agricultural crop straw feed utilization in China. Trans. Chin. Soc Agric. Eng. 32, 1–9. doi: 10.11975/j.issn.1002-6819.2016.22.001

[B9] Ferreira G. Teets C. (2017). Effect of planting density on yield, nutritional quality, and ruminal *in vitro* digestibility of corn for silage grown under on-farm conditions. Prof. Anim. Sci. 33, 420–425. doi: 10.15232/pas.2017-01621

[B10] Fu R. (2008). Recommendations for the sustainable development of animal husbandry in China. J. Henan Agric. Sci. 37, 137–138. doi: 10.3969/j.issn.1004-3268.2008.12.042

[B11] Gao J. F. (2006). Experimental guidance of plant physiology (Beijing: Higher Education Press).

[B12] Ge J. Z. Zha J. Liang Q. Zhang Y. Ma Z. Q. (2019). Interactive effects of nitrogen and density on silage yield and quality of DeMeiYa 1. Hans J. Soil Sci. 7, 286–291. doi: 10.12677/hjss.2019.7403

[B13] Hashida Y. Kadoya S. Okamura M. Sugimura Y. Hirano T. Hirose T.. (2018). Characterization of sugar metabolism in the stem of Tachisuzuka, a whole-crop silage rice cultivar with high sugar content in the stem. Plant Prod. Sci. 21, 233–243. doi: 10.1080/1343943X.2018.1461016

[B14] Hou W. F. Khan M. R. Zhang J. L. Lu J. W. Ren T. Cong R. H.. (2019). Nitrogen rate and plant density interaction enhances radiation interception, yield and nitrogen use efficiency of mechanically transplanted rice. Agric. Ecosyst. Environ. 269, 183–192. doi: 10.1016/j.agee.2018.10.001

[B15] Huang J. W. Wu J. Y. Chen H. F. Zhang Z. X. Fang C. X. Shao C. H.. (2022). Optimal management of nitrogen fertilizer in the main rice crop and its carrying-over effect on ratoon rice under mechanized cultivation in Southeast China. J. Integr. Agric. 21, 351–364. doi: 10.1016/S2095-3119(21)63668-7

[B16] Jian Y. W. Zhao J. Zhang P. H. (2024). Introduction to factors affecting rice straw silage quality and assessment system. China Dairy Cattle. 3, 5–9. doi: 10.19305/j.cnki.11-3009/s.2024.03.002

[B17] Jiang P. Xu F. X. Liu M. Xiong H. Zhang L. Zhu Y. C.. (2019). Effects of nitrogen management and plant density on grain yield and quality of mid-season hybrid rice in rice-ratoon rice system. China Rice. 25, 37–43. doi: 10.3969/j.issn.1006-8082.2019.03.008

[B18] Li J. (2014). Effects of nitrogen rate and plant density on the quality of foodstuff maize under completely mulched alternating narrow and wide ridges with furrow planting (Lanzhou, Gansu: Master Dissertation of Gansu Agricultural University).

[B19] Li Y. (2020). Effects of planting densities and nitrogen application on yield, quality, water-nitrogen utilization of silage maize in Hexi region (Lanzhou, Gansu: Master Dissertation of Lanzhou University).

[B20] Li G. H. Hu Q. Q. Shi Y. G. Cui K. H. Nie L. X. Huang J. L.. (2018). Low nitrogen application enhances starch−metabolizing enzyme activity and improves accumulation and translocation of non−structural carbohydrates in rice stems. Front. Plant Sci. 9. doi: 10.3389/fpls.2018.01128, PMID: 30108604 PMC6079283

[B21] Liu A. Li Y. W. Yang L. B. Zhang Y. Y. Liao S. P. Li X. K. (2024). Applying nitrogen fertilizer improves the indica rice (*Oryza sativa L.*) quality by coordinating enzyme activity and grain-filling rate. Cereal Chem 101, 1043–1054. doi: 10.1002/cche.10806

[B22] Liu F. C. Sun Y. T. (2008). Studies on the relationship among farmland decrease and land loss of farmers and economic growth. Resour. Sci. 30, 52–57. doi: 10.3321/j.issn:1007-7588.2008.01.008

[B23] Luo Y. S. (2007). Effects of amount of nitrogen applied and planting density on grain yield of II Youhang1 using as ratoon rice cultivar. Chin. Agric. Sci. Bull. 23, 203–206. doi: 10.3969/j.issn.1000-6850.2007.07.048

[B24] Luo Z. Z. Ye T. L. Xu M. G. Xiao X. P. Tan B. Gong C. Y.. (2005). Countermeasures and problems on stockbreeding of agriculture regions in South China. Res. Agric. Mod. 26, 401–406. doi: 10.3969/j.issn.1000-0275.2005.06.001

[B25] Lv Z. L. Zhong S. Q. Yang H. (2010). Study on ratoon rice high and stable yield cultivation techniques. J. Anhui Agric. Sci. 38, 8886–8888. doi: 10.3969/j.issn.0517-6611.2010.17.021

[B26] Nakano H. Hattori I. Morita S. (2019). Dry matter yield response to seeding rate and row spacing in direct-seeded and double-harvested forage rice. Jpn. Agric. Res. Q. 53, 255–264. doi: 10.6090/jarq.53.255

[B27] Nakano H. Morita S. (2008). Effects of time of first harvest, total amount of nitrogen, and nitrogen application method on total dry matter yield in twice harvesting of rice. Field Crops Res. 105, 40–47. doi: 10.1016/j.fcr.2007.07.002

[B28] Nakano H. Morita S. Kitagawa H. Takahashi M. (2009). Effects f cutting height and trampling over stubbles of the first crop on dry matter yield in twice harvesting of forage rice. Plant Prod. Sci. 12, 124–127. doi: 10.1626/pps.12.124

[B29] Sakai M. Iida S. Maeda H. Sunohara Y. Nemoto H. Imbe T. (2003). New rice varieties for whole crop silage use in Japan. Breed. Sci. 53, 271–275. doi: 10.1270/jsbbs.53.271

[B30] Shi P. T. Luo Y. C. Qin Y. Y. Li H. Liang H. H. Lin Y.. (2023). Effects of Cd stress on accumulation of Cd and mineral elements in Xiangliangyou 900 and its rationing rice. Shandong Agric. Sci. 55, 127–133. doi: 10.14083/j.issn.1001-4942.2023.12.017

[B31] Tang R. Z. Liang C. N. Jia C. X. Wei F. W. Liu Y. G. (2014). Different cultivation densities’ impact on the yields of first season rice and ratoon rice. J. Guangxi Agric. 29, 3–6. doi: 10.3969/j.issn.1003-4374.2014.01.002

[B32] Wang Y. C. (2019). Effects of nitrogen management on yield formation of ratoon rice and the related mechanism (Wuhan, Hubei: Doctoral Dissertation of Huazhong Agricultural University).

[B33] Wang Y. C. Li X. F. Lee T. Peng S. B. Dou F. (2021). Effects of nitrogen management on the ratoon crop yield and head rice yield in South USA. J. Integr. Agric. 20, 1457–1464. doi: 10.1016/S2095-3119(20)63452-9

[B34] Wu J. Z. Duan X. J. Yao X. Liu Q. M. Xiao R. P. Zhang X. W.. (2023). Key cultivation techniques and benefit analysis of forage-ratoon rice cropping system. South China Agric. 17, 176–179. doi: 10.19415/j.cnki.1673-890x.2023.07.041

[B35] Wu Z. X. Wu Z. N. (2013). Effect of planting density on II You Hang 2 ratoon rice under throw-transplanting cultivation. J. Zhejiang Agric. Sci. 8, 944–948. doi: 10.3969/j.issn.0528-9017.2013.08.009

[B36] Wu Y. F. Yang S. Y. Miao J. L. Xia Y. L. He Y. F. Shi X. F.. (2024). Effects of mowing time of rice in main season on yield, quality and economic benefit of forage-grain dual-purpose ratoon rice. J. South. Agric. 55, 57–67. doi: 10.3969/j.issn.2095-1191.2024.01.006

[B37] Wu Y. S. Yao X. Y. Cao G. J. Chen C. L. Xiong Y. H. Yin J. H. (2019). Analysis of yield formation and comparison of grain quality of fine quality ratoon rice. Hybrid Rice. 34, 57–63. doi: 10.16267/j.cnki.1005-3956.20190111.010

[B38] Xiong H. Ran M. L. Xu F. X. Hong S. (2000). Achievements and development of rationing rice in South of China. Acta Agron. Sin. 3, 297–304. doi: 10.3321/j.issn:0496-3490.2000.03.008

[B39] Yang D. Chen H. F. Zhuo C. Y. Lin W. X. (2009). Effect of different N application modes in the first cropping rice on the physiobiochemistry of the first cropping rice and its ratoon rice. Chin. J. Eco-Agric. 17, 643–646. doi: 10.3724/SP.J.1011.2009.00643

[B40] Yang C. Zheng C. Yuan K. Xu L. Peng S. B. (2022). Effect of fertilizer management on the yield and quality of different rice varieties in ratoon rice. Chin. J. Rice Sci. 36, 65–76. doi: 10.16819/j.1001-7216.2022.210315

[B41] Yuan S. Cassman K. G. Huang J. L. Peng S. B. Grassini P. (2019). Can ratoon cropping improve resource use efficiencies and profitability of rice in central China. Field Crops Res. 234, 66–72. doi: 10.1016/j.fcr.2019.02.004, PMID: 31007365 PMC6472545

[B42] Zhang W. J. Duan X. J. Yao X. Liu Q. M. Xiao R. P. Zhang J. W.. (2023a). Key cultivation technologies and economic evaluation of the forage-ratoon rice cropping system. South China Agric. 17, 176–179. doi: 10.19415/j.cnki.1673-890x.2023.07.041

[B43] Zhang W. J. Duan X. J. Yao X. Liu Q. M. Xiao R. P. Zhang X. W.. (2023b). Effects of mowing time of first season on silage yield, quality and ratoon rice yield. Crop Res. 37, 110–115.

[B44] Zhang P. H. He J. H. Wang J. Q. Liu C. G. (2008b). The nutrition value forage rice and its utilization on livestock and poultry. China Anim. Husbandry Vet. Med. 35, 17–21.

[B45] Zhang J. G. Liu X. D. Cao Z. Z. Yu Z. Lu Y. G. (2008a). Current status and perspectives of research and utilization of forage rice. Acta Pratacult. Sin. 17, 151–155. doi: 10.3321/j.issn:1004-5759.2008.05.022

[B46] Zhang Q. Liu X. C. Yu G. L. Feng D. Q. Zhao H. Y. Li P.. (2020). Optimal sowing date and stubble height for high-efficiency planting mode of both grain and forage yield of rice in Southern Henan rice growing area. Hybrid Rice. 35, 46–50. doi: 10.16267/j.cnki.1005-3956.20191127.271

[B47] Zhang Y. P. Zhu D. F. Xiong H. Chen H. Z. Xiang J. Lin X. Q. (2012). Development and transition of rice planting in China. Agric. Sci. Technol. 6, 1270–1276. doi: 10.3969/j.issn.1009-4229-B.2012.06.027

[B48] Zheng J. S. Lin W. X. Li Y. Z. Jiang Z. W. Zhuo C. Y. (2004). Nitrogen uptake and grain yield effects of double-cropping rice at different nitrogen application rates in the first crop of ratoon rice. Chin. J. Eco-Agric. 12, 78–82.

