# Supplementary material for: Strategies for enhancing yield and quality of forage-grain dual-purpose ratoon rice: role of first-season density and nitrogen management
Source: Front Plant Sci. 2025 Aug 15;16:1650539. doi: 10.3389/fpls.2025.1650539 (PMC12395572; doi:10.3389/fpls.2025.1650539)
Supplement: Supplementary file 1 [file DataSheet1.docx]

**Table S1** Soil properties of the top soil layer (0-0.20 m) at the experimental sites.

| **Year** | **Organic matter**  **(g kg^-1^)** | **Total N**  **(g kg^-1^)** | **Total P**  **(g kg^-1^)** | **Total K**  **(g kg^-1^)** | **Available N**  **(mg kg^-1^)** | **Available P**  **(mg kg^-1^)** | **Available K**  **(mg kg^-1^)** | **pH** |
| --- | --- | --- | --- | --- | --- | --- | --- | --- |
| 2022 | 30.53 | 1.64 | 0.39 | 17.32 | 116.25 | 10.20 | 144.11 | 6.30 |
| 2023 | 31.28 | 1.63 | 0.39 | 17.15 | 115.31 | 10.40 | 143.41 | 6.28 |

N, P, and K represent nitrogen, phosphorus and potassium, respectively.

**
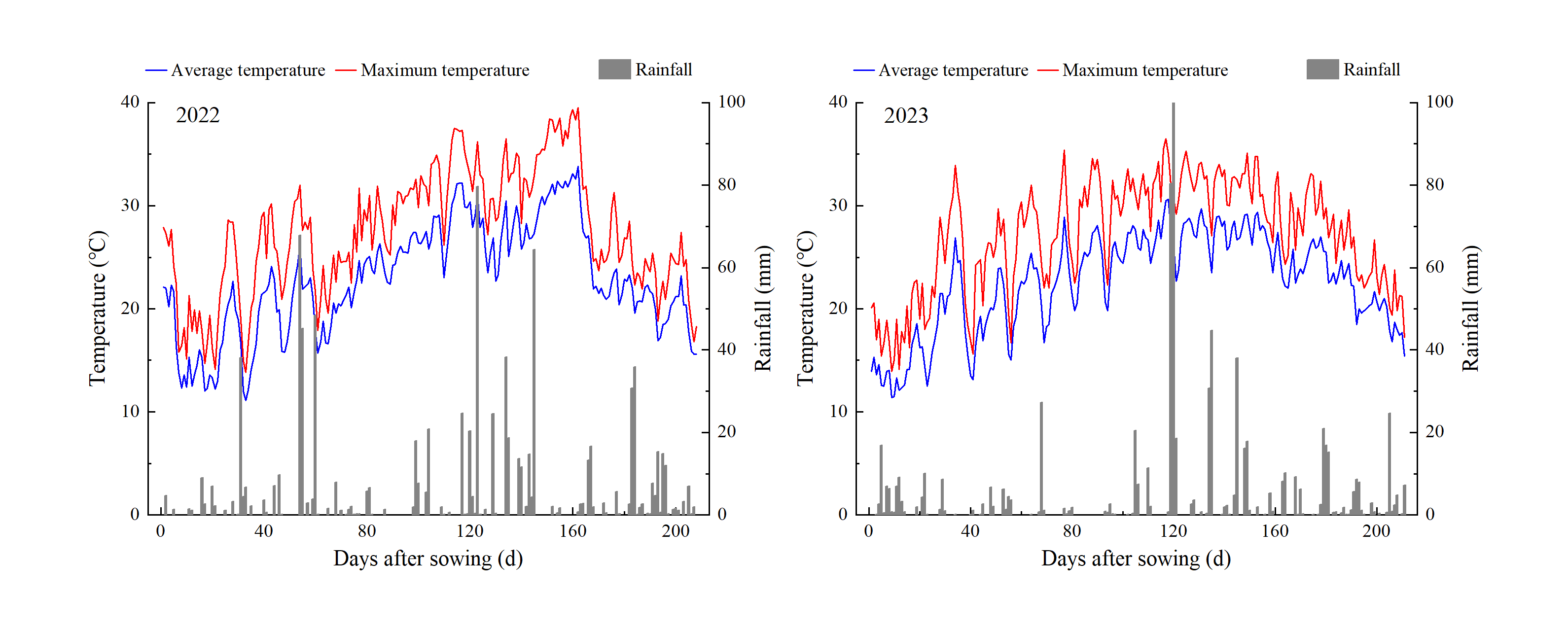
**

**Figure S1** Climate data during the experimental periods.

**Table S2** Analysis of variance on silage rice yield, LAI and SPAD value.

| **Treatment** | **Silage rice yield** | | **LAI** | | **SPAD** | |
| --- | --- | --- | --- | --- | --- | --- |
|  | FY498 | CKYSM | FY498 | CKYSM | FY498 | CKYSM |
| D | 20.512** | 10.526** | 41.003** | 239.649** | 0.818nd | 29.774** |
| N | 16.041** | 40.960** | 14.341** | 92.149** | 10.129** | 14.016** |
| Y | 55.900** | 11.109** | 47.142** | 84.306** | 56.492** | 44.039** |
| D × N | 2.188nd | 1.368nd | 1.158nd | 14.339** | 1.528nd | 1.548nd |

D, N and Y represent planting density, nitrogen application rate and year, respectively. ANOVA *p* values and symbols were defined as: * *p* < 0.05; ** *p* < 0.01; ns: *p* > 0.05, ns means non-significant. The data presented are the mean ± standard deviation, *n* = 3.

**Table S3** Analysis of variance on dry matter accumulation.

| **Growth stage** | **Treatment** | **Stem-sheath** | | **Leaf** | | **Panicle** | | **Total** | |
| --- | --- | --- | --- | --- | --- | --- | --- | --- | --- |
|  |  | FY498 | CKYSM | FY498 | CKYSM | FY498 | CKYSM | FY498 | CKYSM |
| Full heading stage | D | 72.451** | 77.225** | 125.243** | 81.027** | 55.348** | 103.667** | 146.439** | 216.382** |
|  | N | 21.952** | 21.649** | 8.158** | 26.283** | 49.499** | 57.472** | 51.257** | 74.613** |
|  | Y | 1.518ns | 4.867* | 737.513** | 2042.975** | 537.134** | 514.895** | 292.790** | 533.005** |
|  | D × N | 1.333ns | 3.956* | 1.460ns | 0.709ns | 16.870** | 8.277** | 3.500* | 7.378** |
| Maturity stage | D | 23.505** | 150.276** | 23.555** | 130.222** | 115.868** | 110.810** | 159.479** | 275.622** |
|  | N | 36.286** | 12.192** | 10.850** | 30.118** | 34.738** | 36.290** | 76.832** | 54.691** |
|  | Y | 203.671** | 155.787** | 253.916** | 1392.526** | 389.625** | 356.331** | 762.232** | 779.916** |
|  | D × N | 1.311ns | 1.839ns | 1.920ns | 4.861** | 8.090** | 1.499ns | 3.686** | 3.192* |

D, N and Y represent planting density, nitrogen application rate and year, respectively. ANOVA *p* values and symbols were defined as: * *p* < 0.05; ** *p* < 0.01; ns: *p* > 0.05, ns means non-significant. The data presented are the mean ± standard deviation, *n* = 3.

**Table S4-1** RVA profile characters of FY498 in 2022.

| **Year** | **Treatment** | | **PV (RVU)** | **TV (RVU)** | **BV (RVU)** | **FV (RVU)** | **SV (RVU)** | **PeT (min)** | **PaT (℃)** |
| --- | --- | --- | --- | --- | --- | --- | --- | --- | --- |
| 2022 | D1 | N1 | 249.92±4.91a | 157.42±6.35a | 92.50±1.52a | 289.44±4.80a | 39.53±4.20a | 6.07±0.07a | 77.40±0.43a |
|  |  | N2 | 246.60±7.75a | 156.67±4.64a | 89.93±4.71a | 287.70±4.79ab | 41.10±4.43a | 6.00±0.13a | 76.87±0.03a |
|  |  | N3 | 244.92±7.17ab | 155.36±3.70a | 89.56±7.00a | 287.22±1.30ab | 42.30±7.37a | 6.05±0.20a | 77.27±0.46a |
|  |  | Mean | 247.14±3.01A | 156.48±1.68A | 90.66±1.41A | 288.12±0.66A | 40.98±2.37A | 6.04±0.07A | 77.18±0.26A |
|  | D2 | N1 | 245.69±1.90a | 156.23±4.84a | 89.47±4.75a | 287.34±5.31ab | 41.65±4.33a | 6.11±0.17a | 77.45±0.44a |
|  |  | N2 | 241.11±8.17abc | 156.11±0.61a | 85.00±7.72a | 284.39±9.21abc | 43.28±2.40a | 6.08±0.02a | 76.92±0.08a |
|  |  | N3 | 240.11±1.45abc | 152.00±4.87ab | 88.11±6.32a | 283.70±6.51abc | 43.59±6.45a | 6.13±0.07a | 77.17±0.38a |
|  |  | Mean | 242.30±2.13A | 154.78±0.34A | 87.53±2.01A | 285.14±2.00B | 42.84±2.16A | 6.11±0.08A | 77.18±0.25A |
|  | D3 | N1 | 239.53±9.34abc | 151.06±5.74ab | 88.47±4.31a | 281.31±2.23abc | 41.78±8.84a | 6.00±0.07a | 76.95±0.00a |
|  |  | N2 | 233.75±1.18bc | 146.22±1.59bc | 87.53±1.83a | 277.89±4.96bc | 44.13±4.13a | 6.18±0.14a | 77.40±0.87a |
|  |  | N3 | 230.16±4.45c | 142.97±3.42c | 87.19±3.34a | 275.58±2.57c | 45.42±2.57a | 6.13±0.07a | 77.13±0.45a |
|  |  | Mean | 234.48±3.47B | 146.75±1.93B | 87.73±1.74A | 278.26±1.39C | 43.78±4.05A | 6.10±0.03A | 77.16±0.44A |

D1, D2 and D3 refer to the different planting density treatments (16.7 × 10^4^, 20.8 × 10^4^ and 27.8 × 10^4^ hills ha^-1^, respectively). N1, N2 and N3 refer to the different nitrogen fertilizer treatments (150, 225 and 300 kg ha^-1^, respectively). PV, TV, BV, FV, SV, PeT and PaT represent peak viscosity, trough viscosity, breakdown viscosity, final viscosity, setback viscosity, peak time and pasting temperature, respectively. Different lowercase letters followed the values in the same column mean the significant difference of the different combined application of D and N levels at *p* < 0.05. Different uppercase letters mean the significant difference of different average D levels at *p* < 0.05. The data presented are the mean ± standard deviation, *n* = 3.

**Table S4-2** RVA profile characters of FY498 in 2023.

| **Year** | **Treatment** | | **PV (RVU)** | **TV (RVU)** | **BV (RVU)** | **FV (RVU)** | **SV (RVU)** | **PeT (min)** | **PaT (℃)** |
| --- | --- | --- | --- | --- | --- | --- | --- | --- | --- |
| 2023 | D1 | N1 | 314.00±12.14a | 188.44±12.05a | 125.56±5.50a | 355.72±12.88a | 41.72±2.00f | 6.02±0.14a | 81.83±1.93b |
|  |  | N2 | 283.17±3.19b | 171.31±10.42bc | 111.86±8.99b | 348.36±7.85ab | 65.19±5.46de | 5.62±0.17b | 80.70±0.85bcd |
|  |  | N3 | 251.64±7.72ef | 161.97±7.01c | 89.67±7.87cd | 328.72±10.73cd | 77.08±5.49abc | 5.98±0.25a | 79.10±0.80cd |
|  |  | Mean | 282.94±5.18A | 173.91±3.96A | 109.03±3.25A | 344.27±6.98A | 61.33±4.11B | 5.87±0.11A | 80.54±1.09B |
|  | D2 | N1 | 276.86±3.14bc | 177.56±4.75ab | 99.31±7.89c | 337.72±6.89bc | 60.86±4.79e | 5.91±0.03a | 78.65±1.78d |
|  |  | N2 | 259.22±8.25de | 168.72±10.9bc | 90.50±3.91cd | 328.05±3.94cd | 68.83±6.46bcde | 5.93±0.07a | 76.30±1.20e |
|  |  | N3 | 244.58±5.90fg | 161.39±9.74c | 83.20±5.75de | 324.61±12.62cd | 80.03±7.76ab | 5.89±0.03ab | 79.65±0.61bcd |
|  |  | Mean | 260.22±3.63B | 169.22±8.17AB | 91.00±4.67B | 330.13±3.56B | 69.90±2.34AB | 5.91±0.04A | 78.20±0.45C |
|  | D3 | N1 | 264.89±16.77cd | 168.72±10.9bc | 96.17±7.21c | 332.75±14.9bc | 67.86±6.4cde | 6.00±0.07a | 79.92±1.40bcd |
|  |  | N2 | 242.31±2.31fg | 162.06±5.54c | 80.25±6.82de | 315.11±10.53d | 72.80±10.67abcd | 5.98±0.10a | 81.03±0.75bc |
|  |  | N3 | 233.36±6.30g | 157.20±4.88c | 76.16±2.01e | 314.47±5.78d | 81.11±0.55a | 5.78±0.25ab | 85.10±0.96a |
|  |  | Mean | 246.85±7.02C | 162.66±3.10B | 84.19±4.79B | 320.78±8.63C | 73.93±4.37A | 5.92±0.06A | 82.02±0.29A |
|  | D |  | 60.868** | 12.524** | 24.789** | 24.210** | 8.041** | 0.932ns | 21.092** |
|  | N |  | 60.946** | 14.195** | 19.954** | 12.036** | 21.051** | 0.651ns | 6.130** |
|  | Y |  | 148.312** | 82.399** | 13.155** | 598.706** | 261.301** | 23.049** | 163.456** |
|  | D × N |  | 2.395ns | 0.292ns | 1.602ns | 0.817ns | 1.611ns | 2.795* | 9.673** |

D1, D2 and D3 refer to the different planting density treatments (16.7 × 10^4^, 20.8 × 10^4^ and 27.8 × 10^4^ hills ha^-1^, respectively). N1, N2 and N3 refer to the different nitrogen fertilizer treatments (150, 225 and 300 kg ha^-1^, respectively). PV, TV, BV, FV, SV, PeT and PaT represent peak viscosity, trough viscosity, breakdown viscosity, final viscosity, setback viscosity, peak time and pasting temperature, respectively. Different lowercase letters followed the values in the same column mean the significant difference of the different combined application of D and N levels at *p* < 0.05. Different uppercase letters mean the significant difference of different average D levels at *p* < 0.05. ANOVA *p* values and symbols were defined as: * *p* < 0.05; ** *p* < 0.01; ns: *p* > 0.05, ns means non-significant. The data presented are the mean ± standard deviation, *n* = 3.

**Table S5-1** RVA profile characters of CKYSM in 2022.

| **Year** | **Treatment** | | **PV (RVU)** | **TV (RVU)** | **BV (RVU)** | **FV (RVU)** | **SV (RVU)** | **PeT (min)** | **PaT (℃)** |
| --- | --- | --- | --- | --- | --- | --- | --- | --- | --- |
| 2022 | D1 | N1 | 211.03±2.59a | 123.75±3.99a | 87.27±5.99a | 251.28±5.09a | 40.25±7.47c | 6.09±0.10ab | 84.13±1.22a |
|  |  | N2 | 203.86±1.67b | 117.39±1.02b | 86.47±0.95a | 246.14±2.09b | 42.28±3.03bc | 5.95±0.04cd | 81.98±0.51b |
|  |  | N3 | 199.08±3.43c | 114.72±4.7bc | 84.36±1.33a | 243.06±2.60b | 43.97±0.91abc | 5.98±0.10bcd | 81.25±0.44bc |
|  |  | Mean | 204.66±1.51A | 118.62±1.62A | 86.04±1.77A | 246.82±1.23A | 42.17±1.79B | 6.01±0.08A | 82.46±0.52A |
|  | D2 | N1 | 201.39±1.79bc | 115.28±1.47bc | 86.11±2.84a | 243.94±2.41b | 42.56±3.84bc | 6.11±0.03a | 80.42±0.45c |
|  |  | N2 | 192.33±1.74d | 113.56±0.32bcd | 78.78±1.85b | 236.58±0.72cd | 44.25±1.44abc | 5.91±0.03d | 77.55±1.39e |
|  |  | N3 | 187.19±2.72e | 109.70±2.75cd | 77.50±2.46b | 231.61±1.49de | 44.42±2.97abc | 5.95±0.04cd | 80.45±0.43c |
|  |  | Mean | 193.64±1.76B | 112.84±0.66B | 80.79±1.12B | 237.38±0.22B | 43.74±1.78B | 5.99±0.01A | 79.47±0.56B |
|  | D3 | N1 | 191.42±2.53d | 113.56±5.88bcd | 77.86±3.80b | 237.67±3.01c | 46.25±0.6abc | 5.96±0.08cd | 80.22±0.46cd |
|  |  | N2 | 184.08±1.40e | 110.06±1.76cd | 74.03±3.09bc | 231.70±1.35de | 47.61±2.7ab | 6.11±0.08a | 78.88±0.93de |
|  |  | N3 | 178.53±4.65f | 108.45±0.96d | 70.08±3.69c | 227.83±4.13e | 49.30±2.44a | 6.04±0.08abc | 82.28±0.78b |
|  |  | Mean | 184.68±1.68C | 110.69±2.40B | 73.99±3.07C | 232.40±1.42C | 47.72±0.53A | 6.04±0.05A | 80.46±0.30B |

D1, D2 and D3 refer to the different planting density treatments (16.7 × 10^4^, 20.8 × 10^4^ and 27.8 × 10^4^ hills ha^-1^, respectively). N1, N2 and N3 refer to the different nitrogen fertilizer treatments (150, 225 and 300 kg ha^-1^, respectively). PV, TV, BV, FV, SV, PeT and PaT represent peak viscosity, trough viscosity, breakdown viscosity, final viscosity, setback viscosity, peak time and pasting temperature, respectively. Different lowercase letters followed the values in the same column mean the significant difference of the different combined application of D and N levels at *p* < 0.05. Different uppercase letters mean the significant difference of different average D levels at *p* < 0.05. The data presented are the mean ± standard deviation, *n* = 3.

**Table S5-2** RVA profile characters of CKYSM in 2023.

| **Year** | **Treatment** | | **PV (RVU)** | **TV (RVU)** | **BV (RVU)** | **FV (RVU)** | **SV (RVU)** | **PeT (min)** | **PaT (℃)** |
| --- | --- | --- | --- | --- | --- | --- | --- | --- | --- |
| 2023 | D1 | N1 | 223.11±10.88a | 136.44±7.49a | 86.67±3.91a | 289.97±0.30cd | 66.86±8.01c | 4.49±0.36b | 84.35±1.26bc |
|  |  | N2 | 214.75±8.07ab | 132.78±10.25ab | 81.97±3.61ab | 286.30±1.19cd | 71.55±4.00bc | 5.80±0.23a | 81.25±1.17d |
|  |  | N3 | 201.33±3.04bcd | 122.50±8.46abc | 78.83±5.51bc | 282.58±1.50bc | 81.25±0.15ab | 5.87±0.07a | 86.68±1.11a |
|  |  | Mean | 213.06±0.94A | 130.57±3.77A | 82.49±4.16A | 286.29±0.90AB | 73.22±1.37B | 5.38±0.10B | 84.09±0.48A |
|  | D2 | N1 | 208.42±3.54bc | 132.42±4.34abc | 76.00±3.46bcd | 282.86±1.34d | 74.44±2.80abc | 5.76±0.21a | 82.77±0.28cd |
|  |  | N2 | 204.19±7.00bc | 131.17±10.42abc | 73.03±4.1cd | 280.64±0.16bc | 76.45±2.46abc | 5.87±0.00a | 85.92±0.81ab |
|  |  | N3 | 186.00±9.29ef | 122.50±8.46abc | 63.50±1.56e | 269.45±0.60cd | 83.44±3.42a | 5.75±0.20a | 84.23±0.98bc |
|  |  | Mean | 199.54±2.37B | 128.69±1.24A | 70.84±1.40B | 277.65±0.36B | 78.11±2.63AB | 5.79±0.01A | 84.31±0.68A |
|  | D3 | N1 | 195.22±5.29cde | 122.39±5.28abc | 72.83±6.28cd | 276.20±1.59bc | 80.98±5.55ab | 5.78±0.16a | 87.82±0.80a |
|  |  | N2 | 188.33±6.60def | 119.67±5.47bc | 68.67±4.84de | 269.58±1.34ab | 81.25±1.40ab | 5.69±0.10a | 78.95±1.60e |
|  |  | N3 | 178.75±4.40f | 116.56±7.61c | 62.19±5.14e | 263.03±1.10a | 84.28±10.15a | 5.82±0.04a | 87.88±0.55a |
|  |  | Mean | 187.43±5.33C | 119.54±2.91B | 67.90±2.78B | 269.60±1.31A | 82.17±1.13A | 5.76±0.09A | 84.88±0.33A |
|  | D |  | 81.903** | 10.714** | 62.041** | 42.305** | 11.480** | 11.186** | 9.680** |
|  | N |  | 43.658** | 8.111** | 23.995** | 19.953** | 7.793** | 11.028** | 53.122** |
|  | Y |  | 15.261** | 52.684** | 43.677** | 790.048** | 723.131** | 83.153** | 198.202** |
|  | D × N |  | 0.277ns | 0.463ns | 0.842ns | 0.518ns | 0.642ns | 12.111** | 17.201** |

D1, D2 and D3 refer to the different planting density treatments (16.7 × 10^4^, 20.8 × 10^4^ and 27.8 × 10^4^ hills ha^-1^, respectively). N1, N2 and N3 refer to the different nitrogen fertilizer treatments (150, 225 and 300 kg ha^-1^, respectively). PV, TV, BV, FV, SV, PeT and PaT represent peak viscosity, trough viscosity, breakdown viscosity, final viscosity, setback viscosity, peak time and pasting temperature, respectively. Different lowercase letters followed the values in the same column mean the significant difference of the different combined application of D and N levels at *p* < 0.05. Different uppercase letters mean the significant difference of different average D levels at *p* < 0.05. ANOVA *p* values and symbols were defined as: * *p* < 0.05; ** *p* < 0.01; ns: *p* > 0.05, ns means non-significant. The data presented are the mean ± standard deviation, *n* = 3.

**Table S6-1** Initial eigenvalues and variance proportions for the principal components of the standardized data of FY498.

| **Component** | **Initial eigenvalue** | | | **Extracting squared sum load** | | |
| --- | --- | --- | --- | --- | --- | --- |
|  | Total | Variance (%) | Cumulative variance (%) | Total | Variance (%) | Cumulative variance (%) |
| 1 | 7.669 | 58.997 | 58.997 | 7.669 | 58.997 | 58.997 |
| 2 | 3.257 | 25.075 | 84.072 | 3.257 | 25.075 | 84.072 |
| 3 | 1.466 | 11.278 | 95.349 | 1.466 | 11.278 | 95.349 |
| 4 | 0.392 | 2.998 | 98.347 |  |  |  |
| 5 | 0.091 | 0.697 | 99.045 |  |  |  |
| 6 | 0.070 | 0.541 | 99.586 |  |  |  |
| 7 | 0.046 | 0.357 | 99.943 |  |  |  |
| 8 | 0.007 | 0.057 | 100 |  |  |  |
| 9 | 3.954E-16 | 3.041E-15 | 100 |  |  |  |
| 10 | 1.584E-16 | 1.218E-15 | 100 |  |  |  |
| 11 | 1.059E-17 | 8.144E-17 | 100 |  |  |  |
| 12 | -1.890E-16 | -1.454E-15 | 100 |  |  |  |
| 13 | -6.599E-16 | -5.046E-15 | 100 |  |  |  |

**Table S6-2** Component matrix and component score coefficient matrix for the three components extracted according to initial eigenvalues and variance of FY498.

| **Index** | **Component matrix** | | | **Component score coefficient matrix** | | |
| --- | --- | --- | --- | --- | --- | --- |
|  | Component | | | Component | | |
|  | 1 | 2 | 3 | 1 | 2 | 3 |
| Ratoon rice yield | -0.936 | -0.268 | 0.046 | -0.122 | -0.082 | 0.031 |
| Milled rice rate | 0.591 | 0.764 | -0.209 | 0.077 | 0.235 | -0.143 |
| Head milled rice rate | 0.412 | 0.728 | -0.468 | 0.054 | 0.223 | -0.32 |
| Chalkiness rate | 0.956 | -0.26 | -0.059 | 0.125 | -0.080 | -0.040 |
| Chalkiness degree | 0.937 | -0.254 | 0.189 | 0.122 | -0.078 | 0.129 |
| Peak viscosity | 0.989 | -0.100 | 0.054 | 0.129 | -0.031 | 0.037 |
| Breakdown viscosity | 0.969 | 0.007 | 0.169 | 0.126 | 0.002 | 0.115 |
| Setback viscosity | 0.928 | -0.26 | 0.198 | 0.121 | -0.080 | 0.135 |
| Total starch content | -0.100 | -0.815 | 0.453 | -0.013 | -0.250 | 0.309 |
| Protein content | 0.095 | 0.824 | 0.495 | 0.012 | 0.253 | 0.337 |
| Cadmium content | 0.395 | 0.528 | 0.688 | 0.052 | 0.162 | 0.469 |
| Silage rice yield | -0.79 | 0.441 | 0.386 | -0.103 | 0.135 | 0.264 |
| Relative feed value | 0.951 | -0.214 | -0.137 | 0.124 | -0.066 | -0.093 |

**Table S6-3** The comprehensive scores and rankings of FY498 under different planting density and nitrogen application rate.

| **Treatment** | **Score** | **Ranking** | **Component** | | |
| --- | --- | --- | --- | --- | --- |
|  |  |  | 1 | 2 | 3 |
| D1N1 | 1.344 | 1 | 1.926 | 0.147 | 0.963 |
| D1N2 | 0.646 | 2 | 0.802 | 0.763 | -0.429 |
| D1N3 | 0.497 | 3 | 0.077 | 1.860 | -0.339 |
| D2N1 | -0.083 | 4 | 0.529 | -1.343 | -0.484 |
| D3N1 | -0.235 | 5 | -0.208 | -1.041 | 1.411 |
| D2N2 | -0.437 | 6 | -0.105 | -0.765 | -1.448 |
| D2N3 | -0.531 | 7 | -0.739 | 0.154 | -0.966 |
| D3N3 | -0.584 | 8 | -1.420 | 0.568 | 1.230 |
| D3N2 | -0.617 | 9 | -0.863 | -0.342 | 0.062 |

**Table S7-1** Initial eigenvalues and variance proportions for the principal components of the standardized data of CKYSM.

| **Component** | **Initial eigenvalue** | | | **Extracting squared sum load** | | |
| --- | --- | --- | --- | --- | --- | --- |
|  | Total | Variance (%) | Cumulative variance (%) | Total | Variance (%) | Cumulative variance (%) |
| 1 | 7.702 | 59.244 | 59.244 | 7.702 | 59.244 | 59.244 |
| 2 | 3.021 | 23.235 | 82.479 | 3.021 | 23.235 | 82.479 |
| 3 | 1.303 | 10.025 | 92.504 | 1.303 | 10.025 | 92.504 |
| 4 | 0.553 | 4.257 | 96.761 |  |  |  |
| 5 | 0.223 | 1.715 | 98.476 |  |  |  |
| 6 | 0.126 | 0.968 | 99.445 |  |  |  |
| 7 | 0.054 | 0.417 | 99.862 |  |  |  |
| 8 | 0.018 | 0.138 | 100 |  |  |  |
| 9 | 2.154E-16 | 1.657E-15 | 100 |  |  |  |
| 10 | 8.015E-17 | 6.166E-16 | 100 |  |  |  |
| 11 | -1.197E-17 | -9.204E-17 | 100 |  |  |  |
| 12 | -2.542E-16 | -1.956E-15 | 100 |  |  |  |
| 13 | -6.034E-16 | -4.642E-15 | 100 |  |  |  |

**Table S7-2** Component matrix and component score coefficient matrix for the three components extracted according to initial eigenvalues and variance of CKYSM.

| **Index** | **Component matrix** | | | **Component score coefficient matrix** | | |
| --- | --- | --- | --- | --- | --- | --- |
|  | Component | | | Component | | |
|  | 1 | 2 | 3 | 1 | 2 | 3 |
| Ratoon rice yield | -0.848 | 0.018 | 0.243 | -0.110 | 0.006 | 0.187 |
| Milled rice rate | 0.411 | 0.793 | 0.395 | 0.053 | 0.263 | 0.303 |
| Head milled rice rate | 0.101 | 0.971 | 0.171 | 0.013 | 0.321 | 0.131 |
| Chalkiness rate | 0.973 | -0.127 | 0.048 | 0.126 | -0.042 | 0.037 |
| Chalkiness degree | 0.906 | -0.380 | -0.045 | 0.118 | -0.126 | -0.034 |
| Peak viscosity | 0.996 | 0.034 | 0.000 | 0.129 | 0.011 | 0 |
| Breakdown viscosity | 0.972 | 0.091 | -0.069 | 0.126 | 0.030 | -0.053 |
| Setback viscosity | 0.971 | 0.021 | 0.077 | 0.126 | 0.007 | 0.059 |
| Total starch content | 0.813 | -0.097 | 0.537 | 0.106 | -0.032 | 0.412 |
| Protein content | -0.218 | 0.845 | -0.433 | -0.028 | 0.280 | -0.333 |
| Cadmium content | -0.419 | -0.465 | 0.617 | -0.054 | -0.154 | 0.474 |
| Silage rice yield | -0.779 | 0.429 | 0.423 | -0.101 | 0.142 | 0.325 |
| Relative feed value | 0.814 | 0.392 | 0.088 | 0.106 | 0.130 | 0.067 |

**Table S7-3** The comprehensive scores and rankings of CKYSM under different planting density and nitrogen application rate.

| **Treatment** | **Score** | **Ranking** | **Component** | | |
| --- | --- | --- | --- | --- | --- |
|  |  |  | 1 | 2 | 3 |
| D1N1 | 0.990 | 1 | 1.539 | 0.180 | -0.378 |
| D1N2 | 0.839 | 2 | 1.074 | 0.837 | -0.543 |
| D1N3 | 0.358 | 3 | 0.089 | 1.301 | -0.236 |
| D2N1 | 0.216 | 4 | 0.729 | -1.445 | 1.040 |
| D2N2 | 0.184 | 5 | 0.118 | 0.071 | 0.836 |
| D2N3 | -0.292 | 6 | -0.909 | 0.354 | 1.851 |
| D3N2 | -0.493 | 7 | -0.778 | 0.357 | -0.778 |
| D3N1 | -0.772 | 8 | -0.315 | -1.792 | -1.110 |
| D3N3 | -1.031 | 9 | -1.547 | 0.137 | -0.682 |
